# Mechanical hierarchy in the formation and modulation of cortical folding patterns

**DOI:** 10.1038/s41598-023-40086-9

**Published:** 2023-08-14

**Authors:** Poorya Chavoshnejad, Liam Vallejo, Songyao Zhang, Yanchen Guo, Weiying Dai, Tuo Zhang, Mir Jalil Razavi

**Affiliations:** 1 Department of Mechanical Engineering, Binghamton University, Binghamton, NY 13902 USA; 2 Brain Decoding Research Center and School of Automation, Northwestern Polytechnical University, Xi’an, 710072 Shaanxi China; 3 Department of Computer Science, Binghamton University, Binghamton, NY USA

**Keywords:** Mechanical engineering, Biophysics, Computational models

## Abstract

The important mechanical parameters and their hierarchy in the growth and folding of the human brain have not been thoroughly understood. In this study, we developed a multiscale mechanical model to investigate how the interplay between initial geometrical undulations, differential tangential growth in the cortical plate, and axonal connectivity form and regulate the folding patterns of the human brain in a hierarchical order. To do so, different growth scenarios with bilayer spherical models that features initial undulations on the cortex and uniform or heterogeneous distribution of axonal fibers in the white matter were developed, statistically analyzed, and validated by the imaging observations. The results showed that the differential tangential growth is the inducer of cortical folding, and in a hierarchal order, high-amplitude initial undulations on the surface and axonal fibers in the substrate regulate the folding patterns and determine the location of gyri and sulci. The locations with dense axonal fibers after folding settle in gyri rather than sulci. The statistical results also indicated that there is a strong correlation between the location of positive (outward) and negative (inward) initial undulations and the locations of gyri and sulci after folding, respectively. In addition, the locations of 3-hinge gyral folds are strongly correlated with the initial positive undulations and locations of dense axonal fibers. As another finding, it was revealed that there is a correlation between the density of axonal fibers and local gyrification index, which has been observed in imaging studies but not yet fundamentally explained. This study is the first step in understanding the linkage between abnormal gyrification (surface morphology) and disruption in connectivity that has been observed in some brain disorders such as Autism Spectrum Disorder. Moreover, the findings of the study directly contribute to the concept of the regularity and variability of folding patterns in individual human brains.

## Introduction

Throughout the development of the human brain, the initially small and smooth-surfaced brain undergoes a transformation known as cortical folding. During this transformation, the brain experiences a volumetric expansion followed by the formation of gyri (convex folds) and sulci (concave folds) on the cortical surface^1,2^. These folds are commonly classified into one of three categories: primary, secondary, or tertiary folds. Primary folds are the first to form and are the largest and deepest. Secondary and tertiary folds are developed shortly after by subdividing the primary folds^3,4^. Research has shown that, in terms of their location, primary folds are relatively consistent across individuals of the same species, while secondary and tertiary folds are highly variable^3,5^. These folds significantly impact brain function; abnormalities in cortical folding patterns have been linked to cognitive impairments as well as neurodevelopmental and psychiatric disorders such as autism spectrum disorder (ASD), epilepsy, and schizophrenia^6,7^. The cortex, the outer layer of the brain, also called gray matter, is made up of cell bodies and capillaries. The white matter, or subcortex, is a complex system of axonal fibers that act as the connections between different areas of the brain. These fibers can be classified into one of three categories^8–10^: projection, commissural, or association fibers. Projection fibers extend radially from the subcortical regions to the cortex. Commissural fibers connect the two hemispheres of the brain through commissures. Association fibers connect different regions located in the same hemisphere^2^. The location and orientation of these fibers change throughout the development process, which led to many studies that explored the relationship between them and cortical folding patterns^2,11–16^. Initially, it was speculated that the axonal fibers pulled on the cortex and thus caused cortical folding^17,18^. However, this theory has been largely disregarded due to contradicting evidence^19^. In fact, the folding cortex pulls on the axons to trigger axonal elongation and white matter growth rather than axons pulling on the brain to induce cortical folding^14,20^.

Currently, the leading theory for the mechanism of cortical folding is differential tangential growth (DTG), which has been supported by recent experimental and computational findings^21–27^. This theory suggests that cortical folding is attributed to mechanical instability arising from either the differential tangential growth within the cortical layer^25,28^ or the radial differential tangential expansion due to faster growth of the cortical plate compared to the subcortical white matter^23,29,30^. The difference in the growth rates creates compressive stresses in the cortex, which eventually induces buckling^30–32^. By using DTG theory, some classical brain disorders associated with the morphogenesis of the developing brain have been modeled and explained by mechanical instability and post-perturbation concepts^33,34^. Sophisticated computational modeling based on DTG has begun to emerge as a powerful tool to validate or verify the cortical folding results from experiments. Recently, nonlinear finite element (FE) analysis has offered valuable insight into the growth, morphology, and function of the brain^23,24,34–41^. The results of those studies suggest that DTG is the potential driving force for cortical folding. However, this driving force in idealized brain models produces random patterns, which are highly dependent on the initial conditions and imperfections^26,42,43^.

Although it is widely accepted that the differential growth initiates folding, studies have shown that many other parameters, such as axonal connectivity and patterned growth, play a role in the final morphology of the brain^2,3,14–16,18,22,34,39,44–46^. The literature shows that the emergence and development of brain connectivity mainly occur simultaneously with cortical folding during the fetal stage^11,47^. A connection between abnormal folding patterns of the brain and underlying connectivity has been observed in several brain disorders such as ASD^48–52^, schizophrenia^53–58^, bipolar disorder^59,60^, as well as alteration in both domains in polymicrogyria^61–63^ and lissencephaly^64,65^. It is postulated that two morphometric domain alterations in ASD have an early onset, possibly during the fetal stage of neurodevelopment^66,67^. For example, in ASD brains, it has been shown that reduced gyrification is linked to reduced connectivity^48^ or, in some cases, to an increased local connectivity and a reduced distant connectivity^49^. However, the underlying mechanics and mechanisms of this correlation are not known. We previously have shown that the interplay between DTG and axonal fibers plays a role on the modulation of cortical folding patterns^2^. We observed that the convolution of the cortex pulls areas with a high concentration of axonal fibers toward gyri rather than sulci. Despite these findings, there is still limited knowledge on how the location and shape of fully folded gyri and sulci are determined in mature brains. Moreover, the important contributors and their hierarchy in the formation and modulation of folding patterns remain poorly understood. This knowledge gap in the interplay of brain morphology and other determinant factors, such as brain connectivity, is a barrier to discovering the origin of brain neurodevelopmental disorders.

In this study, we develop a multiscale mechanical model to explore and elucidate the hierarchy between DTG, initial geometrical undulations, and axonal connectivity in the formation and modulation of folding patterns. This study reveals the competition and correlation between important factors in determination of location and shape of fully folded gyri and sulci. Moreover, we explain how 3-hinge gyral folds form in the human brain and how important mechanical factors in brain folding control their locations and shapes. A 3-hinge gyral fold, hereafter 3-hinge fold, is the conjunction of gyrus crest lines from three different orientations^68^. In contrast to ordinary gyri, gyral hinges are of importance because they have the thickest cortices, the strongest long-range axonal connections, the most pronounced connective diversity, and the most aggregative functional profiles^43,69–73^. This study will be a foundation to explore the mechanics of the correlation between abnormal gyrification and brain dysconnectivity in some brain disorders such as ASD.

## Material and methods

### Growth model

A quarter of a sphere that includes gray matter, white matter, and axonal fiber bundles was created to study the effect of initial undulations and axonal fibers on brain morphology after growth and folding. The undulations mimic the initial surface irregularities of the brain in the fetal stage and unfolded condition. The white matter, the core of the sphere, is surrounded by gray matter with a thickness of 1.5 mm in the model^30,74^. The white matter is composed of the Extracellular Matrix (ECM) and axonal fiber bundles. The human brain contains trillions of axonal fibers with a total length reaching hundreds of thousands of kilometers. As a result, modeling individual axonal fibers in a macroscopic growth model presents significant challenges, and achieving a complete representation of such complexity is currently considered extremely challenging. However, the distribution of axonal fibers in the white matter follows a highly organized pattern. These fibers tend to aggregate into specific bundles or tracts, each following distinct trajectories and pathways. Therefore, we offer the concept of "axonal fiber bundles" to capture the contribution of axonal fibers to gyrification in our study. These bundles represent groups of axonal fibers that are aligned and packed together within specific locations and orientations. By defining axonal fibers as bundles, we aimed to incorporate the heterogeneous and anisotropic nature of the white matter substrate into our models, considering its essential role in the gyrification process. Through the control of bundle density, our models also enabled the representation of regions with varying densities of axonal fibers, mirroring the distribution observed in the real human brain's white matter^75^.

In this study, we considered the theory of differential growth as the driving force of gyrification. In the field of continuum mechanics, the deformation map $\boldsymbol{\varphi }$ is introduced to describe the mapping of a material particle from its original position ${\varvec{X}}$ in the undeformed (reference) configuration ${\mathcal{B}}_{0}$ to its new position ${\varvec{x}}=\boldsymbol{\varphi }\left({\varvec{X}}\right)$ in the deformed (spatial) configuration ${\mathcal{B}}_{t}$. Furthermore, the deformation gradient ${\varvec{F}}={\nabla }_{\mathbf{X}}\boldsymbol{\varphi }$ is introduced to quantify the mapping of the line element from the reference to spatial configuration, while the Jacobian *J* is used to characterize the local volume alteration from the reference to spatial configuration. Both the deformation gradient ${\varvec{F}}$ and Jacobian *J* can be further decomposed multiplicatively into an elastic part and a growth part^76^.

1 $$\begin{array}{*{20}c} {{\varvec{F}} = {\varvec{A}} \cdot {\varvec{G}}\quad {\text{and}}\quad J = {\text{det}}\left( {\varvec{F}} \right) = J^{e} J^{g} } \\ \end{array}$$

where **A** and ${\varvec{G}}$ are elastic and growth deformation tensor, respectively. ${J}^{e}$ and ${J}^{g}$ are Jacobian corresponding to the elastic and growth tensor, with the relation described as ${J}^{e}=\mathrm{det}({\varvec{A}})$, ${J}^{g}=\mathrm{det}({\varvec{G}})$. The growth of tissues, including the gray matter, white matter, and axonal fiber bundles, was mimicked by a relative thermal expansion, which has been shown to realistically simulate biological tissue growth^77–79^. Imaging data shows that the thickness of gray matter remains relatively constant during brain development^30,74,80^, which indicates that gray matter grows mainly transversally isotropic^81–84^. Therefore, a uniform transversal growth with the growth tensor ${G}_{c}={g}_{c}{\varvec{I}}+\left(1-{g}_{c}\right) {\widehat{{\varvec{n}}}}_{s}\otimes {\widehat{{\varvec{n}}}}_{s}$ was applied to the gray matter. In the equation, ${g}_{c}$ (${g}_{c}$ > 1) is a constant scaler, ***I*** is the unit tensor, and ${\widehat{{\varvec{n}}}}_{s}$ is the normal vector of the surface^77,78^. It is worth noting that a homogeneous tangential growth of the cortex was assumed in the models for the purpose of simplification. However, in reality, the growth of the cortical plate is heterogeneous in both tangential and radial direction^28,85,86^. In contrast, we assumed that the growth of the ECM followed an isotropic expansion, as defined by ${G}_{W}={g}_{w}{\varvec{I}}$. Additionally, the growth of axonal fiber bundles was assumed to occur along the axis of the bundles (modeled by truss elements). The isotropic and transversal growth were simulated using isotropic and transversal thermal coefficients in Abaqus FE software^87^. The gray and white matter growth rates were extracted from Andescavage et al.^88^ in the range between 25 and 40 GWs^89^. We introduce the metric of the whole model growth ratio as the third root of the volume of the grown model over the initial volume of the model to describe the growth process, encompassing both gray and white matter. In the growth model, we initiated the simulation by replicating the same volumes of the gray and white matter in a spherical setup, using ¼ of the volumes to represent ¼ of a sphere with symmetric boundary conditions. Subsequently, the gray matter was assigned tangential growth, while the white matter underwent isotropic growth, aiming to achieve the volumes observed at the second time point.

A neo-Hookean hyperelastic material model was employed in the study to replicate the mechanical response of gray and white matter when subjected to quasi-static deformation^2,16,77^. The model utilized a strain energy density function defined by Eq. 2:

2 $$U={C}_{10}\left({\lambda }_{1}^{2}+{\lambda }_{2}^{2}+{\lambda }_{3}^{2}-3\right)+\frac{1}{{D}_{1}}{({J}^{el}-1)}^{2}$$

where ${C}_{10}$ and ${D}_{1}$ are material parameters, ${\lambda }_{i}$ dente the principal stretches and ${J}^{el}$ is determinant of the elastic deformation gradient. In the case of axonal fibers, their material properties were also modeled using the neo-Hookean model. However, when considering a truss element, only the principal stretch ${\lambda }_{1}=\lambda$ is taken into account. In the simplified 1D scenario, the principal stretches ${\lambda }_{2}={\lambda }_{3}=1$^90^, which leads to the reduction of Eq. (2) as follows:

3 $$U={C}_{10}\left({\lambda }_{1}^{2}-1\right)+\frac{1}{{D}_{1}}{({J}^{el}-1)}^{2}$$

**Figure 1:**
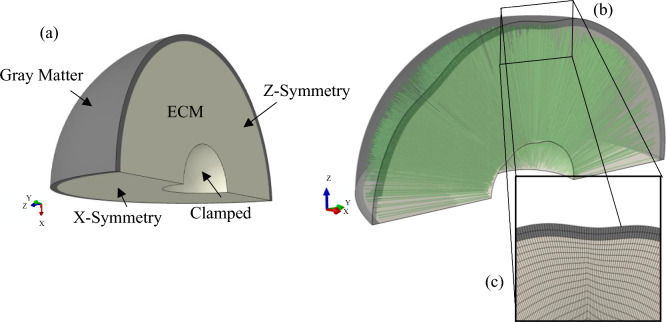
(**a**) Initial geometry of the brain model without surface undulation. White matter part consists of the ECM and axonal fiber bundles. (**b**) The initial geometry of the model after applying undulations. (**c**) Magnified region of a meshed model with a negative (inward) undulation. Axonal fiber bundles are extended from the base of the white matter towards the interface of the white and gray matter.

The shear moduli of gray matter, ECM, and axonal fiber bundles were assigned as 500, 70, and 310 Pa, respectively^91,92^. To focus on the contribution of axonal fibers and geometrical undulation to the folding process, other material and geometrical properties, such as the shear modulus and growth rate of white and gray matter and the gray matter thickness, were assumed to be constant in all models. Figure 1a shows the initial geometry of the quarter-sphere model. Both gray matter and ECM were meshed using an 8-node linear brick (C3D8R), and the truss element (T3D2) was used to mesh axonal fibers, Fig. 1b. To reduce the simulation cost, a small sphere far from the study area was removed from the model and the remaining surface was clamped. Furthermore, the mesh size was gradually increased from the outer surface of the ECM to the center of sphere. A self-contact constraint to avoid the self-penetration of the gray matter during folding was applied on the free surface of the gray matter. Considering the normal vector of each face, a symmetry boundary condition was applied to each cut face of the model. The independence of the results from the boundary conditions and mesh size was checked.

A python code was developed to apply the geometrical undulations to the surface of the gray matter of the meshed model. Figure 1c shows a zoomed-in undulation on the model. Each undulation was applied by displacing the nodes of the meshed model based on a Gaussian distribution. Mapping the surface of the spherical model to a plan surface enabled us to use a 2D Gaussian distribution. To keep the aspect ratio of the elements in an acceptable range, the amplitude of the applied undulations decreases gradually from the outer surface to the center of the sphere. Thus, the outer surface of the gray matter has the maximum amplitude, and the center of the sphere has zero amplitude. Equation (4) describes the Gaussian distribution:

4 $$F=\alpha \mathrm{exp}(-(\frac{{(x-{x}_{0})}^{2}}{2{\sigma }_{x}^{2}}+\frac{{(y-{y}_{0})}^{2}}{2{\sigma }_{y}^{2}}))$$

Here, ${x}_{0}$ and ${y}_{0}$ are the center of the undulation, ${\sigma }_{x}$ and ${\sigma }_{y}$ are the *x* and *y* spread of the blob, and α is the amplitude. The parameters of the Gaussian function allow undulations to be created at random sites with random amplitudes and blob spreads. This study investigated models with 4, 5, or 6 undulations at four different amplitudes (α): 1- without undulation ($\alpha =0$); 2- low amplitude ($\alpha =0.25\times t$); 3- medium amplitude ($\alpha =0.50\times t$); 4- high amplitude ($\alpha =1\times t$), where *t* is the thickness of the gray matter. We categorized the amplitudes of the initial undulations using some preliminary models and according to their effectiveness on the formation of the consistent folding patterns.

**Figure 2:**
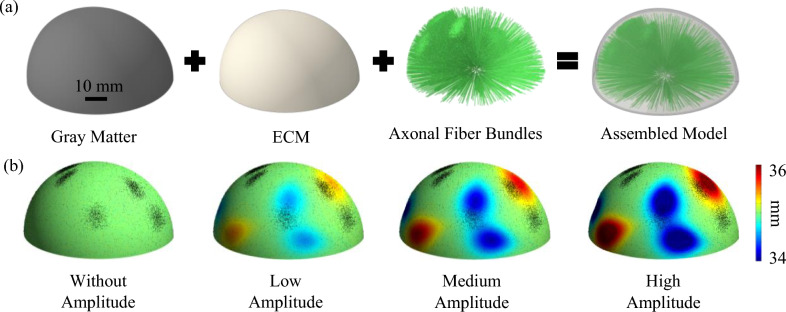
Developing brain growth model including surface undulations and axonal fiber bundles. (**a**) Initial state of a 3D model without undulations. The gray matter, white matter, and axonal fiber bundles are assembled to form a unified model. Transparent colors are used to depict the components of the model in one image. (**b**) An example model with five concentration areas of axonal fiber bundles and five surface undulations, including three negative undulations and two positive undulations with different amplitudes. The gray matter is depicted with the radial distance contour (mm). Red regions illustrate the positive undulations while blue regions have negative undulations. The black dots show the projection of the tip of axonal fiber bundles on the surface of the gray matter for a better representation. To capture the shallow undulations in the figure, we used a contour range of 34–36 mm, which is within the bounds of minimum (33.5 mm) and maximum (36.5 mm) radial distance.

In addition to incorporating geometrical undulations, this study explores the contribution of axonal bundles on brain gyrification. In our previous study, we showed that the axonal bundles could regulate the morphology of the folding^2^. Here, the goal is to investigate the interaction of geometrical undulations and concentrated areas of axonal fiber bundles. Axonal fibers bundles are created inside the ECM and projected from the inner to the outer surface of the ECM by using truss elements. We considered axonal fiber bundles (nerve tract) to be a group of individually aligned axonal fibers with a diameter equal to 250 µm^14,93,94^. The embedded element method^2,95–97^, was used to embed the axonal fibers bundles inside the ECM. In this technique, the translation degrees of freedom of embedded element nodes are confined to the interpolated values of the corresponding degrees of freedom of the host element. Therefore, axonal bundles are bonded to the hosted elements of the ECM. The geometrical undulation code was also applied for all axonal fiber bundles. Therefore, each axonal fiber bundle extends from the clamped surface of the ECM and reaches the interface between ECM and gray matter after applying undulations. According to the imaging data of Dean et al.^98^ for the fetal brains, the volume fraction of axonal bundles inside the ECM was set to 20%. Therefore, axonal bundles were added to the ECM until the volume fraction of axonal bundles reached 20%. Considering the imaging data that shows the density of axonal fibers varies in the white matter and that there are concentrated areas of axonal fibers^2,43,99^, we implemented concentrated areas of axonal bundles into the ECM. To do so, 5% of the white matter is initially filled by axonal fiber bundles using uniform random distribution. Then, the remaining axonal fiber bundles (15% of the white matter) were distributed at the concentrated sites using a normal distribution. To avoid an overlap between concentration areas, a minimum allowable distance between their geometrical centers was set at 15 mm. In respect to the number of concentration areas, three scenarios with 4, 5, and 6 concentration areas were modeled. In addition to the scenarios with concentration areas, one scenario with a uniform random distribution of axonal bundles was also created as a control group. Figure 2 explains the creation of models with different scenarios.

In Fig. 2a, the components of a model which includes gray matter, ECM, and axonal bundles are disassembled to illustrate different sections of the model. The right figure of Fig. 2a depicts a model with transparent colors including axonal fiber bundles (green color) inside the white matter. Figure 2b displays a set of scenarios with the same number of undulations and concentrations but with different amplitudes for undulations. The contour shows the radial distance of the gray matter. Red areas demonstrate the positive (outward) undulations while the blue areas represent negative (inward) undulations. In this specific case, there are two positive undulations and three negative undulations (five undulations in total). Black dots depict the projection of fiber bundles on the surface of the gray matter. From left to right, the amplitude of undulations increases. As mentioned earlier, four amplitude scenarios were considered. For the scenario without amplitude, the contour shows a uniform color equal to the size of the model (35 mm). Moving from the low amplitude to the high amplitude, the color of the undulations changes from light to dark, which shows deeper cavities and more elevated protrusions.

In this study, we considered three different numbers of undulations (4, 5, and 6), four different concentrations (0, 4, 5, and 6), and four amplitude levels ($0\times \mathrm{t}$, $0.25\times \mathrm{t}$, $0.50\times \mathrm{t}$, and $1\times \mathrm{t}$). In total, 48 different scenarios were defined. For each scenario, three different cases were created and studied to reduce the effect of randomness. We introduced a numerical abbreviation for each scenario based on the number of undulations, number of concentrations, and the level of amplitude to present the results in a convenient way. As an example, a scenario with four undulations, four concentrations, and with a low amplitude is represented with 441. Table 1 shows the developed and studied scenarios.

**Table 1:** Labeling different scenarios (48 scenarios) based on the number of undulations, number of concentrations, and amplitude of undulations.

Undulation	Amplitude	0 Concentration	4 Concentrations	5 Concentrations	6 Concentrations
4	$0\times t$	400	440	450	460
	$0.25\times t$	401	441	451	461
	$0.50\times t$	402	442	452	462
	1 $\times t$	403	443	453	463
5	$0\times t$	500	540	550	560
	$0.25\times t$	501	541	551	561
	$0.50\times t$	502	542	552	562
	1 $\times t$	503	543	553	563
6	$0\times t$	600	640	650	660
	$0.25\times t$	601	641	651	661
	$0.50\times t$	602	642	652	662
	1 $\times t$	603	643	653	663

For each scenario, three different cases were developed to reduce the effect of randomness. In total, 144 models were developed and studied (48 × 3 = 144). *t* is the thickness of the gray matter.

### Detection of gyrus, sulcus, and wall

A combination of maximum principal curvatures (MPCs) and gaussian curvatures (GCs) was used to distinguish gyri, walls, and sulci in the folded brain models. The MPC and the GC were computed on the membrane between white and gray matter. Gyral regions were specified as those with MPC $\ge \hspace{0.17em}$0.2 (1/mm) or GCs > 0.2 and sulci regions with MPC $\le \hspace{0.17em}$0.2 and the rest as walls, all the criteria were chosen empirically.

### Local gyrification index

The local gyrification index (LGI), a three-dimensional extension of the gyrification index, is an automated method to study regional changes in gyrification, which enables a non-biased estimation of whole-brain morphometric changes. The LGI was defined as an area ratio between the outer hull and the pial surface of the gray matter^100,101^. The outer hull surface was generated by wrapping a surface around the pial surface of the gray matter, which encloses any gap less than 10 mm. For each vertex on the pial surface, a MATLAB code was developed to calculate the surface area of both the pial surface and hull surface in a radial distance of less than 5 mm in a cylindrical coordinate system that originates at the center of the spherical model. The LGI of each vertex was obtained by dividing the total area of the pial surface by the total area of the hull surface.

### Detection of 3-hinge gyral folds

Gyral net pipeline^102^ was used to extract the number and shape of the 3-hinge folds in the models after folding. Detailed information regarding the extraction of 3-hinge folds can be found in our previous publications^68,103,104^.

### Statistical analysis

In this study, we used the Pearson correlation (PC) and p-value to evaluate the effect of the studied parameters in different scenarios, such as the number of undulations, number of concentrations, and amplitude of undulations, on each calculated factor from the results. To evaluate the strength of correlation, we first considered *p*-value; *p*-values greater than 0.01 are not statistically significant. If the *p*-value between two parameters is less than 0.01, then we use the Pearson correlation. We considered the Pearson correlation |r| value of $\le 0.4$ as a weak correlation, 0.4–0.6 moderate correlation, and $\ge 0.6$ strong correlation.

## Results

Different growth scenarios were developed based on the initial geometry of the gray/white matter and the location of axonal fiber bundles. In general, three scenarios for the geometrical undulations (4, 5, and 6 undulations) and four scenarios for the axonal fiber bundles concentration sites (4, 5, 6, and a uniform random distribution) were considered. Furthermore, four amplitudes for the undulations were applied in the models (without undulation, low amplitude, medium amplitude, and high amplitude). Finally, each case was studied three times to reduce the effect of randomness. In total, 144 growth and folding models were developed and studied (see the Methods section for more details). The presented data in this section is the average of the three cases for each setup.

### Gyrification of the brain model including axonal fiber bundles

**Figure 3:**
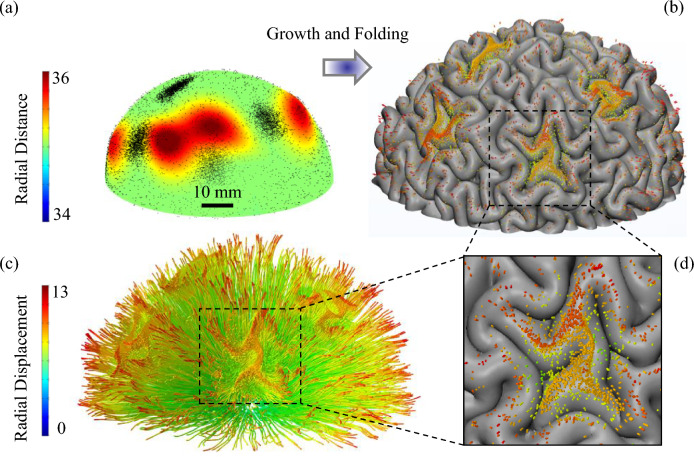
A multiscale mechanical model for the growth and folding of the brain including axonal fiber bundles. (**a**) Initial geometry of the model. The model has 4 undulations and 4 concentration areas of axonal fiber bundles. Twenty percent of the white matter is filled by axonal fiber bundles. The diameter of a bundle is 250 µm. The contour shows the radial distance in the spherical coordinate system. (**b**) Growth and folding of the model. The tips of fiber bundles are projected on the gray matter’s surface to better show their location at the interface of gray-white matter. (**c**) Deformation of axonal fiber bundles inside the white matter after growth and folding of the model. The contour shows the radial displacement in the spherical coordinate system. (**d**) Location of fiber bundles’ tips in a zoomed-in state. Axonal fiber bundles settle mainly in gyri and form 3-hinge patterns in the concentration areas.

Figure 3, as an example, shows the initial and folded morphologies of a model with four positive undulations and four axonal concentration areas. The model before growth and at the initial stage has a smooth surface and positive undulations. At the initial state, the projection of fiber bundles on the surface of the gray matter is illustrated by black dots. The models start to grow and at a certain point of the growth process, a mechanical instability emerges as the result of the mismatch between the growth rates of the gray and white matter. The volume of gray matter increases by 4.5 times while the volume of white matter increases only by 2.7 times during 25–40 gestational weeks^89^. Faster growth of the gray matter induces compressive stresses in the cortical plate and creates instability. Growth ratio, *g,* of the whole model is defined as the third root of the volume of the grown model over the initial volume of the model. At the beginning of growth (g = 1.33), the amplitude of cortical undulations rises, but there is still no sign of folding. After a certain amount of growth (g = 1.33–1.56), the model reaches its unstable point and bifurcates to a new geometry, where peaks and valleys start to form into gyri and sulci. By transitioning into post-instability morphology, the system releases its strain energy^2^, and by further growth (g = 1.56–2.29), the emerged folds become more convoluted and are comparable to a real mature brain. Figure 3b shows the folded morphology of the initial model after growth and folding. The ECM of the white matter is suppressed in Fig. 3c to observe the position of the axonal fiber bundles after folding. In the initial status and before folding (Fig. 3a), the fibers are straight bundles that extend from the inner to the outer surface of the ECM (Fig. 2a). Figure 3b also depicts the projection of the axonal fiber bundles’ tips on the surface of the gray matter after folding. Tracking the tips of fiber bundles and comparing them with the position of gyri and sulci in Fig. 3b,c clearly indicates that the axonal fibers settle mainly in gyri rather than sulci. The main part of the white matter that acts as a substrate for the cortex folding is corona radiata. Corona radiata is a section of white matter that includes several fiber tracts and carries most of the neural traffic to and from the cerebral cortex. In the model, 20% of the white matter volume is occupied with axonal fiber bundles (7,523 counts). The statistical data show that 45.7%, 29.9%, and 24.4% of axonal bundles in this specific case settle in gyri, walls, and sulci, respectively.

**Figure 4:**
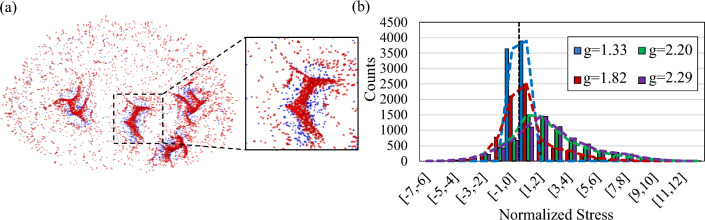
Stress type in axonal fiber bundles. (**a**) Axonal fiber bundles’ tips under tension (red) or under compression (blue). (**b**) Axial stress distribution in the axonal fiber bundles at the interface of the gray and white matter during the folding process in different growth ratios.

Statistical results regarding the distribution of the axial stress in the axonal fiber bundles show that they are mainly under tension during the growth process and after folding. Figure 4a distinguishes fiber tip color based on the type of the experiencing stress (tensile vs. compressive). Red dots depict fiber tips under tension and blue dots show fiber tips in compression at the end of the folding process. Figure 4a clearly shows that the number of fibers under tension is significantly higher than the fibers under compression. The histogram analysis in Fig. 4b also shows the distribution of the normalized axial stress in the fiber tips. Positive or negative stress represents pulling or pushing force, respectively. In all the folding steps, the number of fibers under tension is higher than those under compression. The ratio between the number of fibers under tension to the number of fibers in compression equals 1.07, 1.35, 3.81, and 4.17 for growth ratio 1.33, 1.82, 2.20, and 2.29, respectively. These numbers show that as folding progresses, the total number of fibers under compressive force decreases because it is added to the total number of fibers under tensile force.

### Correlation between the distribution of axonal fiber bundles and folding morphology

**Figure 5:**
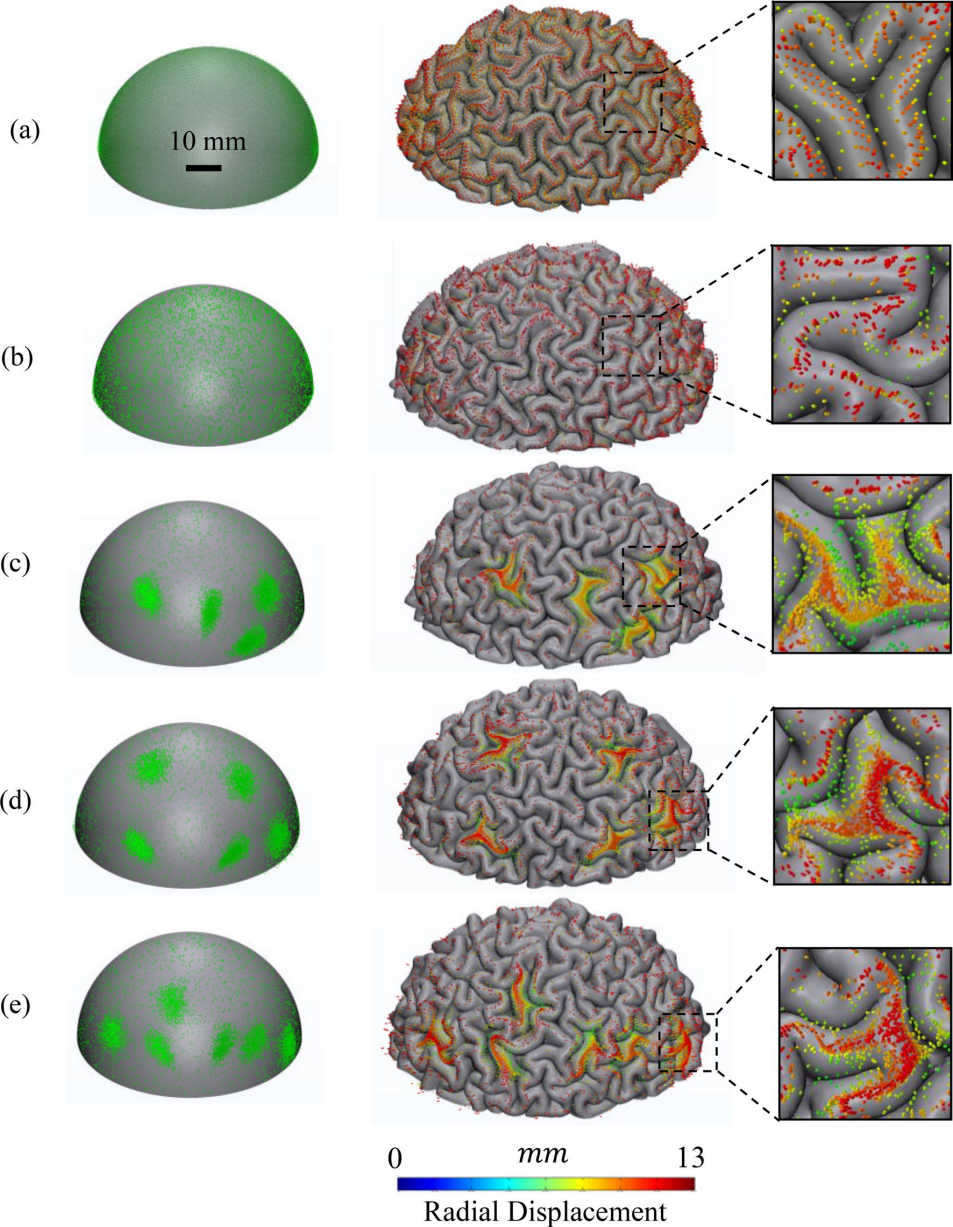
Growth and folding of brain models with a different number of concentration areas. The number of bundles is the same in the models. There are no initial undulations in the models ($\alpha =0$). The locations of the concentration areas are selected randomly. Axonal bundles occupy 20% of the white mater volume. (**a**) Uniform distribution. (**b**) Uniform random distribution. (**c**) Four concentration areas. (**d**) Five concentration areas. (**e**) Jayakumari (Six) concentration areas. First column shows the initial distribution of axonal bundles before folding. Second column shows the projection of axonal bundles on the surface of gray matter after folding. Third column shows the magnification of a concentration area for each case.

Figure 5 summarizes the results of one set of four scenarios with 4, 5, and 6 concentration areas, as well as a model with a uniform distribution and a model with a uniform random distribution of axonal fiber bundles. The first column shows the projection of axonal fiber bundles’ tips on the free surface of each model's gray matter at its initial state. Axonal fiber bundles’ tips are depicted with a radial distance contour. These models show the difference in axonal fiber concentration between each of the scenarios. The second column shows the projection of axonal fiber bundles on the gray matter after folding. In all cases, most of the axonal fiber bundles settle in gyri. For the models including concentration areas (Fig. 5c–e), the path of the crest lines of gyri and the path of the tips of dense axonal fiber bundles are the same (magnified images). Even for the cases with uniform or random uniform distributions, a greater number of axonal fiber bundles are in the gyri than the sulci. The last column shows a magnified image of one concentration area for each case.

**Figure 6:**
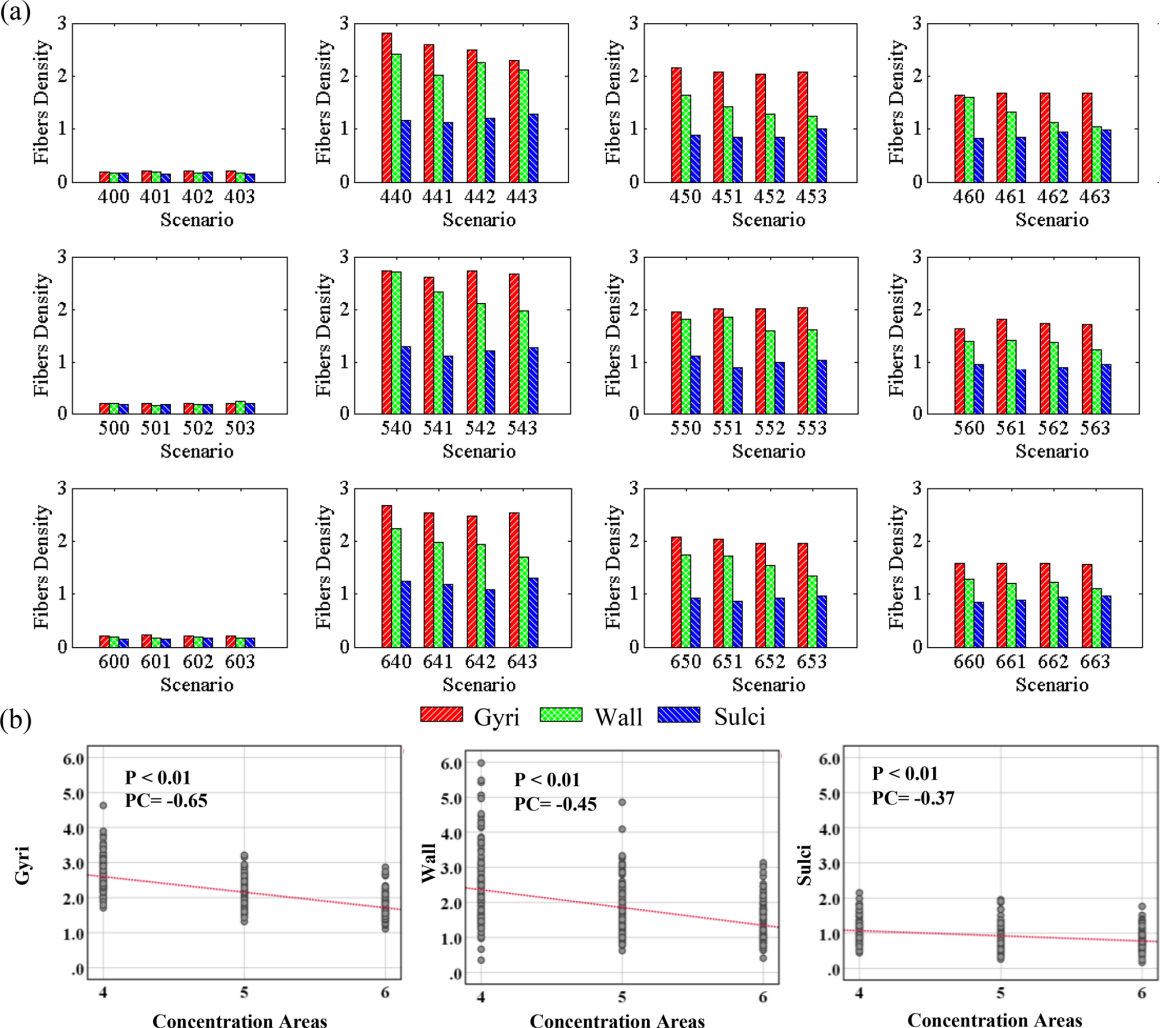
(**a**) Local fiber density in gyri, wall, and sulci after folding in the models with a different number of undulations and concentration areas. The local fiber density refers to the quantity of fibers present within a given unit surface area (in 1 mm^2^). For each combination of undulation and concentration, models without, low, mean, and high amplitude of undulations are compared. The first, second, and third numbers in the scenario labels show the number of undulations, number of concentration areas of axonal fibers, and amplitude of undulations, respectively. See Table 1 for the detail of labeling. Red, green, and blue colors indicate for gyri, wall, and sulci, respectively. (**b**) Correlation analysis of the data for each parameter that has a significant correlation. *P*: P-value, PC: Pearson Correlation.

We ran three different cases for each scenario to reduce the effect of randomness and have meaningful statistical results. The first represented statistical data is the local density of the bundles in gyri, wall, and sulci. To calculate the local density for each concentration area, we considered the fiber bundles and the interface between gray matter and white matter located within a radius of 5 mm from the center of that concentration area. We computed the total surface area of each gyri, wall, and sulci, along with the corresponding number of fiber bundles within each region. The local density was determined by dividing the number of bundles by the surface area of each respective region. The averaged results are presented in Fig. 6. The first column presents scenarios without concentration areas, indicating that the bundles are randomly distributed throughout the white matter. As there are no predefined concentration areas in these scenarios, we randomly selected four distinct points to serve as the centers of concentration areas. As expected, the scenarios without concentration areas exhibit noticeably lower local fiber density. Additionally, in these cases, the ratio between the fiber density in the gyri and the fiber density in the sulci is notably low, specifically with an average ratio of $1.2$. The results for the uniform distribution of fiber bundles were not reported, as they showed the same trend as the uniform random distribution. The observed difference in axonal fiber density between the gyri and sulci can be attributed to the bending of the cortex. This bending leads to compression at the bottom of the gyri and stretching at the bottom of the sulci. Consequently, given the assumption of a uniform random distribution of fibers, the fiber density in the gyri is higher than in the sulci. However, it is important to emphasize that the uniform distribution of axonal fiber bundles does not sufficiently account for their contribution to the formation and modulation of cortical folding patterns. Across all scenarios with concentration areas, the density of bundles in the gyri is notably higher compared to the walls and sulci, aligning with findings from imaging studies. The average ratio between axonal fiber density in the gyri to the sulci is determined to be $2.1$. While the phenomenon of compression and stretch at the bottom of gyri and sulci remains valid, it is evident that other factors must be considered to explain this significant increase in the fiber density. One possible explanation for this can be attributed to the energy minimization of the entire structure, including gray matter, white matter, and axonal fiber bundles during folding. Indeed, due to the normal distribution of bundles within the concentration areas, the axonal fiber density in these regions exhibits variations from the initial stage. Following the folding process, areas with higher fiber density tend to be predominantly located in the gyri. The statistical analysis conducted revealed no significant or moderate correlation between axonal fiber density and the number or amplitude of undulations. However, a strong correlation was observed between axonal fiber density and the number of concentration areas. This correlation is expected since the total number of bundles within concentration areas remains constant.

### Correlation between initial undulations and folding morphology

**Figure 7:**
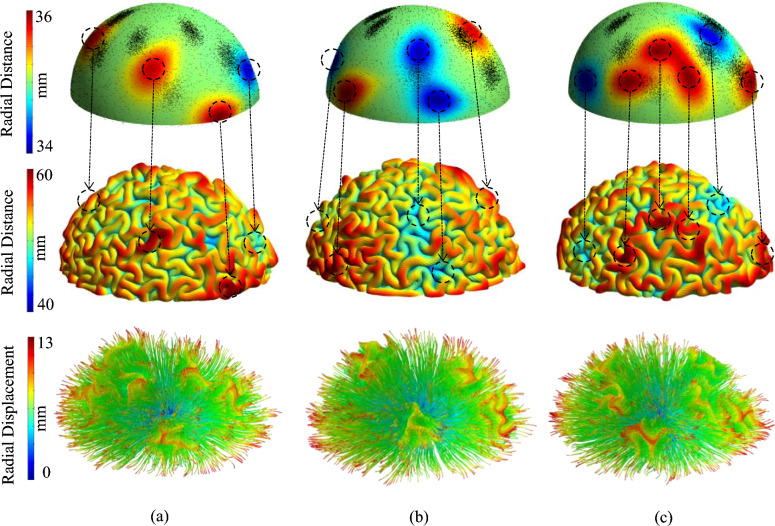
Growth and folding of three models with different numbers of initial undulations. All the undulations have a medium amplitude. (**a**) Four initial undulations (scenario 462). (**b**) Five initial undulations (scenario 552). (**c**) Six initial undulations (scenario 662). The first row shows the initial state of the models. The second row shows the morphology of each model after folding. The contour of the first and second row are the radial distance (mm). The third row shows the deformation of axonal fiber bundles inside the white matter after growth and folding of the model. The contour illustrates the radial displacement (mm).

**Figure 8:**
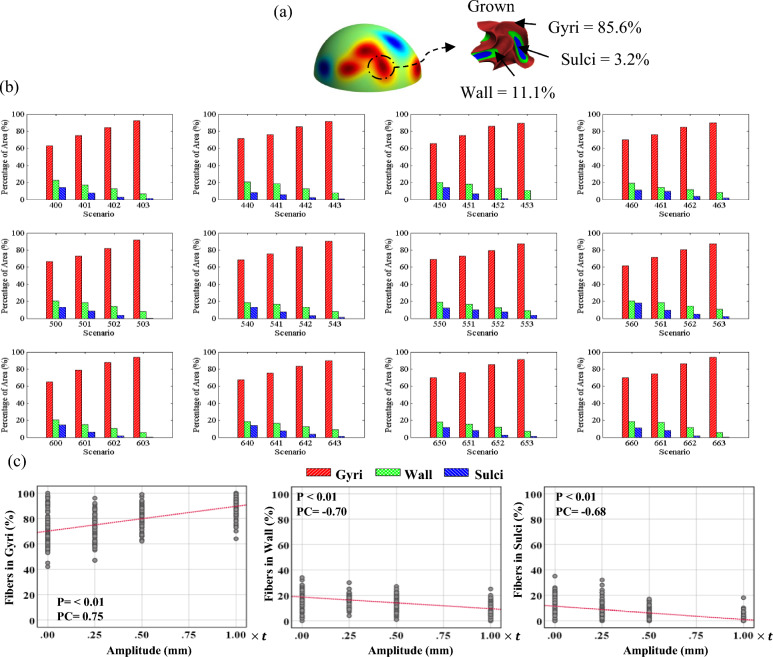
(**a**) A positive undulation before and after growth. (**b**) Percentage of positive undulation area in gyri/wall/sulci after folding in different scenarios. The undulation area in the initial geometry is defined as a circular surface with a radius of 5 mm, in which its center is located at the center of undulation. (**c**) Correlation analysis of the data for each parameter that has a significant correlation. P: P-value, PC: Pearson Correlation.

**Figure 9:**
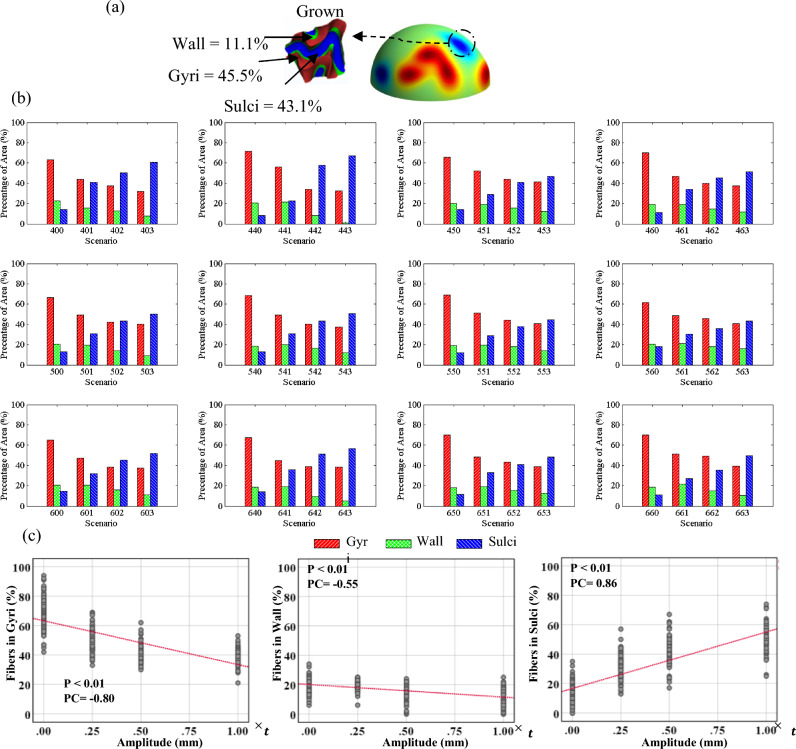
(**a**) A negative undulation before and after growth. (**b**) Percentage of negative undulation area in gyri/wall/sulci after folding in different scenarios. (**c**) Correlation analysis of the data for each parameter that has a significant correlation. P: P-value, PC: Pearson Correlation.

Figure 7 shows the initial state and the folded morphologies of the models with different numbers of undulations. The positive and negative undulations mimic the convex and concave surface irregularities that the fetal brain has in the early stage of development. Figure 7 presents cases with 4, 5, and 6 undulations, all of which have a medium amplitude. The contour shows the deformation in the radial direction. Red areas highlight positive undulations and blue regions indicate negative undulations at the initial state. The projection of axonal fiber bundles is depicted with black dots in initial state. The corresponding final morphology and deformation of axonal fiber bundles of each model are presented below the initial state. As the marked regions show, the negative undulations typically settle in sulci after folding, while the positive undulations settle in gyri. This observation is consistent in all of the presented cases. To quantify this observation, the negative and positive undulations of each scenario were separated and statistically analyzed to better understand and interpret the effect of the parameters. The average results for each scenario are reported in Figs. 8 (for positive undulations) and 9 (for negative undulations), which show what percentage of undulation area in the initial geometry will settle in gyri, walls, or sulci after folding. The undulation area in the initial geometry is defined as a circular surface with a radius of 5 mm centered at the center of undulation. Data in Figs. 8 and 9 were obtained by calculating the surface area of each undulation located in gyri, walls, and sulci after folding (see Fig. 8a).

Figure 8a shows the surface of the initial state of the white matter with the contour of radial distance. The magnified images show one positive and one negative undulation after folding. Red, green, and blue colors in the magnified image illustrate the distinguished gyri, wall, and sulci, respectively. From the image, the percentage of the area identified as gyri for the positive undulation is significantly higher than wall and sulci. However, when a negative undulation is studied, the total area of gyri decreases while the total area of sulci increases significantly. Figure 8b shows the statistical results related to the positive undulations for each scenario. Each surface element of the initial undulation region was traced to see if it is positioned in gyri, walls, or sulci after folding. Each column shows the percentage of the undulation area located in each geometrical feature (gyrus, wall, sulcus). The presented results are obtained by dividing the area of the elements in gyri/walls/sulci after folding by the total area of the undulation before folding. The results of correlation analysis between different parameters are shown in Fig. 8c. Only the parameters that exhibit a significant correlation are displayed in the figure. Refer to the supplemental materials for a detailed analysis of all the parameters. As Fig. 8b clearly shows, the positive undulations in the initial status will form mainly gyri after folding, regardless of the number of undulations, concentration areas, or the amplitude of the undulations. A statistical analysis of Fig. 8b indicates that there is a strong correlation between the amplitude of the initial undulations and the percentage of the area in gyri after folding. In all the scenarios, increasing the amplitude of initial undulations raised the percentage of area in gyri. The correlation coefficient between the amplitude and the percentage of area in gyri is 0.75, which shows a robust positive relationship. The number of undulations and concentration areas do not significantly affect the percentage of area in gyri because the calculated correlation coefficients are 0.04 and 0.007, respectively. Even for the scenarios without undulation, the percentage of area in gyri is higher than wall and sulci. This happens due to the definition of gyri, wall, and sulci. We used the interface between the gray matter and white matter as a surface of interest to find gyri, wall, and sulci. On this surface, each gyrus is a double-layer surface, like a narrow peak, while the sulcus is a wide valley. Therefore, the gyri's total area is higher than the wall and sulci in general. Although the percentage of area in gyri is higher even without undulations, application of undulations increases the percentage of area in gyri significantly. Further details of correlation between initial positive undulations and folding morphology are presented in Table S1.

Figure 9 represents similar results to Fig. 8b, but for the negative undulations. In contrast with the positive undulations, there is a negative relationship between the amplitude of negative undulations and the percentage of the area in gyri. By increasing the amplitude of negative undulations, the percentage of area in gyri decreases significantly. Meanwhile, an increase in the amplitude results in a considerable increase in the percentage of area in sulci. The correlation coefficient obtained between the amplitude of undulations and the percentage of area in gyri is -0.8, but for the percentage of area in sulci, it is 0.86. This means that the probability of formation of sulci in areas with negative undulations is very high and increases when the amplitude of undulation increases. The correlation coefficients between the percentage of surface area in sulci and the number of undulations or number of concentrations demonstrate that there is no meaningful relationship between them. More details of correlation between initial negative undulations and folding morphology can be found in Table S2.

### Correlation between initial undulations and axonal concentration areas with the location of 3-hinge gyral folds

**Figure 10:**
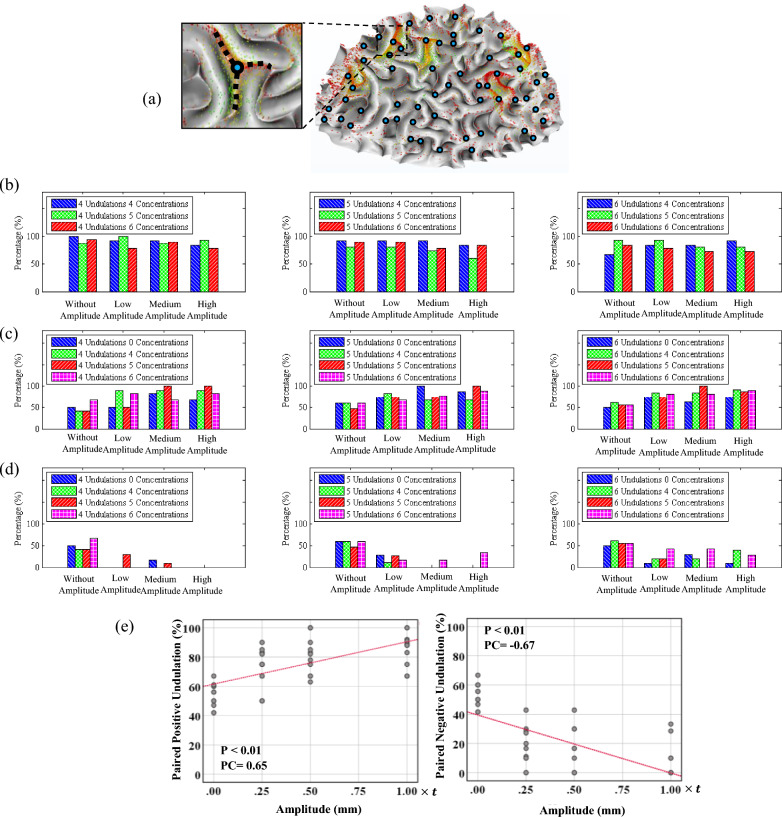
Correlation between the undulations and concentration areas with the formation of 3-hinge folds. (**a**) white matter surface with mapped axonal fiber bundles tips and detected 3-hinge folds. (**b**) Fraction of concentration areas that have a 3-hinge folds in their neighborhood in each scenario. (**c**) Fraction of positive undulations that have a 3-hinge folds in their neighborhood in each scenario. (**d**) Fraction of negative undulations that have a 3-hinge folds in their neighborhood in each scenario. The neighborhood is defined as a 5 mm distance from the center of the undulation or concentration area of axonal fiber bundles. (**e**) Correlation analysis of the data for each parameter that has a significant correlation. P: P-value, PC: Pearson Correlation.

Figure 10 depicts the probability of evolution of 3-hinge folds in the location of concentrations and undulations or their neighborhood. The neighborhood is defined as a 5 mm distance from the center of the undulation or concentration area of axonal fiber bundles. Figure 10a shows a folded white matter and projection of axonal fiber bundles on its surface. Each blue sphere shows the location of one 3-hinge fold in the model. Figure 10a visually shows that in the location of concentration areas, the probability of having a 3-hinge fold is very high. Figure 10b shows the percentage of concentration areas with a corresponding paired of 3-hinge fold in their neighborhood. For instance, for the scenario with 4 undulations, 4 concentration areas, and medium amplitude, 88% of the concentration areas have a paired 3-hinge fold in their neighborhood.

Figure 10 shows a strong relationship between the location of concentration areas and the location of 3-hinge folds. The overall average shows that in the location of more than 84% of the concentration areas, a 3-hinge fold has emerged. Therefore, axonal fiber bundles could be one of the factors responsible for the formation of 3-hinge folds that is consistent with imaging data extracted from the human brain^73^. Further statistical analysis shows that there is a very weak correlation between the number of concentration areas and the fraction of concentration areas that have a corresponding 3-hinge fold pair. The third row of Fig. 10 shows the percentage of the location of positive undulations that have a corresponding 3-hinge fold in their neighborhood. The first column of each chart in the third row shows the chance of having a paired 3-hinge fold without having an undulation. The average data of the results for the cases without undulation indicates that 53% of the searched points have a paired 3-hinge fold. As the correlation coefficient demonstrates (PC = 0.69), there is a strong positive relationship between the amplitude and the percentage of paired 3-hinge folds (68%). Increasing the amplitude of undulations raises the chance of having a paired 3-hinge fold in all scenarios. However, there is no rational correlation between the number of undulations and the percentage of pairs. The bottom row of Fig. 8 illustrates the percentage of correspondence between negative undulations and 3-hinge folds. The first column of each chart in the third row is for the cases without undulations. In contrast to positive undulations, when there are negative undulations, the chance of having a paired 3-hinge fold in the neighborhood area is reduced. The calculated correlation coefficient shows a strong negative relationship (PC = − 0.70). As a result, it can be concluded that there is a high chance of emergence of 3-hinge folds in the neighborhood of positive undulations with high amplitude and, more importantly, around concentration areas of axonal fiber bundles.

### Correlation between initial undulations and axonal concentration areas with local gyrification index

**Figure 11:**
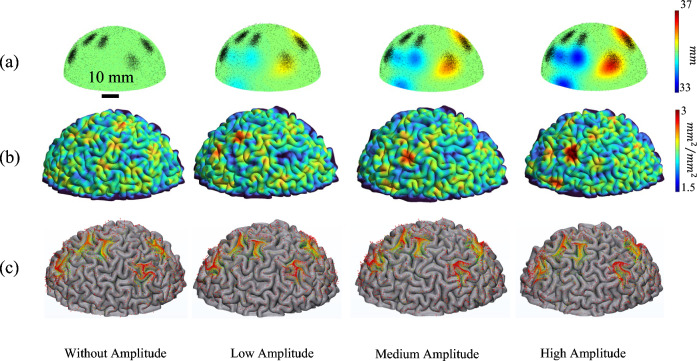
The dependency of LGI to the concentration areas and amplitude of the undulations. (**a**) Initial state of a scenario with 5 concentration areas and 3 negative undulations with different amplitudes. The contour indicates the radial distance (mm). (**b**) The folded result of each case with the contour of local gyrification index. The LGI increases from blue to red. (**c**) Projection of axonal bundles on the surface of gray matter after folding.

Another metric that is used to analyze cortical folding patterns is the local gyrification index (LGI). LGI is a parameter that quantifies the folded brain surface area ratio compared with the surface area of an outer, smooth, wrapped surface^105,106^. A large LGI indicates deeper or more convoluted folds, while a low LGI indicates smooth folds. Figure 11 combines the initial state, final state, and calculated LGI for a scenario with 4 undulations and 5 concentration areas. LGI was calculated by dividing the surface area of the pial surface by the surface area of the hull surface. The second row depicts the initial state of each model with the contour of radial distance. The red areas indicate the positive undulations while blue areas indicate negative undulations of the initial model. The amplitude of the undulations increases from left to right. The third row shows the result of the LGI for each corresponding model after folding. To discover the effect of concentration areas on the LGI, the final state of each model with the projection of axonal fibers on the surface of gray matter is depicted in the last row. An increase in the amplitude of the undulations increases the gap between LGI of areas with negative and positive undulations. Furthermore, the LGI decreases in the areas with the concentration of axonal fiber bundles.

**Figure 12:**
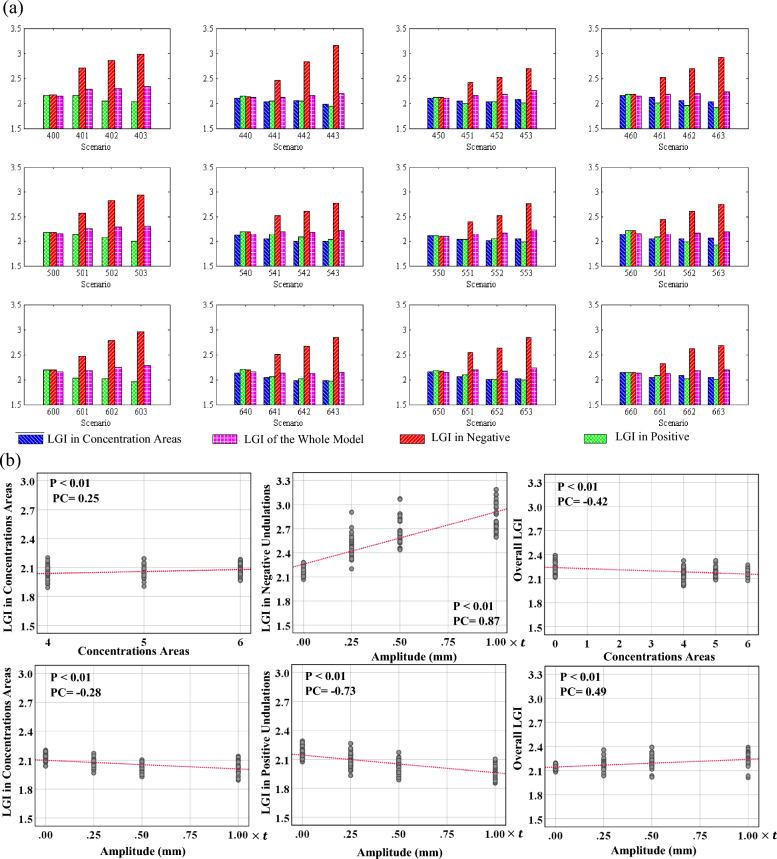
(**a**) Obtained LGI for each scenario in the location of concentration areas and positive/negative undulations. (**b**) Correlation analysis of the data for each parameter that has a significant correlation. P: P-value, PC: Pearson Correlation.

A statistical analysis based on the results of all cases was performed to better understand the correlation between the LGI and the undulations and concentration areas. Figure 12 shows the average data for each scenario. For each scenario, four different LGIs are reported: the average LGI in concentration areas; the average LGI in positive undulations; the average LGI in negative undulations; and the LGI of the whole model. Because the axonal fiber bundles are distributed uniformly in the first column scenarios, the average LGI in the concentration area is eliminated from the charts. Consistent with the results of Fig. 11, the average LGI in the concentration areas of axonal bundles is lower than the average overall LGI in all scenarios. In all scenarios, in the presence of undulations (amplitude $\ne 0$), the average LGI in negative undulations after folding is significantly higher than the other regions of the model. Meanwhile, the positive undulations have the lowest average LGI after folding. The amplitude of the undulations has a strong positive correlation with the LGI in negative undulations (PC = 0.87) and a strong negative correlation with the positive undulations (PC = − 0.73). This result clearly shows that there is a correlation between the connectivity and LGI that has been observed in the image-based studies of neurodevelopmental brain disorders such as ASD. This will be further discussed in the discussion section. Table S3 presents the details of correlation between initial undulations and axonal concentration areas with LGI.

## Discussions

In this study, we established a multiscale finite element model for brain gyrification motivated by the hypothesis that axonal fibers can regulate the formation of a developing brain. The main goal was to understand the mechanical hierarchy in the modulation of cortical folding patterns. From the perspective of mechanics, the results of this study suggested that the formation and regulation of folding patterns of a developing human brain are highly associated with the initial geometry and interplay between differential tangential growth and axonal tension theories.

### Effect of initial geometry in the folding morphology

Our results showed that the locations with positive undulations in the initial geometry would form mainly gyri after folding regardless of the number of undulations, number of concentration areas of axonal fibers, and amplitude of the undulations. However, an increase in the amplitude of a positive undulation increases the chance of forming a gyrus after folding at the same location. In contrast to positive undulations, negative undulations in the initial geometry will form sulci after folding, and any increase in the amplitude of the undulations increases the chance of the formation of sulci. Tracking the formation of sulci and gyri from the early gestational weeks to the postnatal brain in humans also reveals consistent results^107^. Our statistical observations can explain how the initial geometrical undulations of primary folds in the fetal stage after growth and folding form gyri and sulci in the mature brain. A good example is the central gyrus and central sulcus sites in the fetal brain. The central sulcus appears as a small downward point or as a groove in the parasagittal region around 21 GW^107^. We can consider the central sulcus as a large negative undulation at the fetal stage according to its initial depth. As the imaging studies show, after growth and folding, the central sulcus will form a deep, consistent sulcus^107^. Unlike the central sulcus, the precentral sulcus, which can be considered a low-amplitude negative undulation, does not develop a consistent sulcus in individual brains. The precentral sulcus forms anterior and approximately parallel to the central sulcus and has a different shape in individual brains^108^. In our study, we measured the depth of the central sulcus (representing a negative undulation) before and after folding on three individual fetal brains at two longitudinal time points (averaged): 28.86 GW and 36.29 GW. Prior to folding, the depth of the central sulcus had a ratio of 1.15 ± 0.1 mm, while after folding, the depth increased to 9.25 ± 0.5 mm. This results in an approximate ratio of the depth after folding to the depth before folding as 8. To assess the accuracy of our models, we also measured the corresponding depths in the models associated with the 28.86 GW and 36.29 GW time points. Specifically, we determined the ratio of the negative undulation to the depth of the created sulcus for multiple undulations. The observed ratios were 15.8 ± 0.6, 8.3 ± 0.4, and 6.0 ± 0.4 for the low, medium, and high amplitudes, respectively. These results demonstrate a good agreement with the imaging data, which as stated, the ratio is approximately 8 for the central sulcus.

In a real brain, the depth of sulci can vary, while the distance of the gyri from the center remains relatively consistent. We speculate that the same radial distance of gyri in a real brain arises from the constraint of cerebrospinal fluid pressure^41^ and the skull^109^. It has been shown that the skull flattens the crest line of gyri^109^. As the skull is a growing but stiff material compared to brain tissue, it can radially align the crest lines of gyri. However, in our model, there is no skull constraint as it has been demonstrated that cortical folding is not the consequence of the skull constraint. Therefore, the maximum point of positive undulation has a larger radial distance compared to the other formed gyri. In contrast, sulci can penetrate into the soft tissue (white matter) without a rigid barrier similar to the skull. Consequently, the depth of sulci exhibits more variation than the radial distance of gyri.

Positive (upward) or negative (inward) undulations in the initial geometry might be associated with the heterogeneous growth of the cortical plate. We have previously shown that the human brain experiences heterogeneous growth at the cortical plate due to the rate of the migration of neurons^85^. Faster growing local areas typically form positive undulations, and slower growing areas form negative undulations. In this study, we used a uniform cortical thickness with a uniform tangential growth rate. However, positive and negative undulations can resemble the faster and slower growing areas, respectively. In reality, the thickness of the cortical plate and its local growth rates are not uniform. In a study, we showed that the change of the cortical thickness over time on the longitudinal fetal MRI atlas data provides evidence that the areas with a faster growth rate consistently engender gyral regions^85^. From another perspective, we can consider positive undulations as convex patterns with positive maximal principal curvature (MPC) values, whereas negative undulations have negative values. This means that the local initial curvature is a determinant factor in the regulation of folding patterns after folding. Our results also showed that the initial undulations with high amplitudes (positive or negative) are dominant to axonal fiber bundles in the determination of the location of gyri and sulci after folding. However, in the absence of the concentration areas of axonal fibers, differential growth can produce only random folding patterns. It is important to mention that the determination of the location of gyri and sulci is not solely dependent on positive or negative undulations. As suggested by Smart and McSherry^110,111^, the initiation of a gyrus involves local growth in the subplate, while the cortical plate at the future sulcal site remains immature with retarded growth. This observation has been further supported by Borrell and Gotz^86^ and Borrell^112^, emphasizing that cortical folding entails not only outward growth but also the coordinated action of inward fissuring mechanisms. Considering these observations in the context of the real brain, we have previously demonstrated that radially faster growing areas (heterogeneous growth) in the cortical plate also can engender gyrus after folding^85^. This finding aligns with the current study, as local undulations can arise as a result of heterogeneous growth patterns in the cortical plate or subplate. It should be noted that these mechanisms play a role alongside the positive and negative undulations in determining the specific location and patterns of gyri and sulci.

Further statistical analysis showed that an increase in the amplitude of positive initial undulations will reduce the LGI of the positive undulation locations after folding, while an increase in the amplitude of negative initial undulations will increase the LGI of the negative undulation locations. To test this model-based observation, we selected four groups of sulci and gyri at the same locations on one adult and one infant brain and calculated the mean curvatures. The cerebral sulci were found to be more convoluted than the gyri, both early on and after completion of the fold (see Fig. S1 and Table S4). Therefore, we can conclude that the locations with negative undulations after folding are more convoluted and consistent than those with positive undulations.

The high-amplitude negative undulations can be associated with sulcal pits^113,114^ (putative first sulcal fold) in the human brain. It has been shown that the position and spatial variance of sulcal pits demonstrate a similar trend between the fetal and adult brains^114^, which is believed to be the result of the formation of the deepest parts of sulci during the early stage of brain development. More recently, the counterparts of these sulcal pits on gyral regions were found and defined as gyral peaks^115^, which were shown to retain across ages on macaque brains and can be associated with the high-amplitude positive undulations. Those landmarks are consistent and preserve their characteristics during development in contrast to the variable secondary folds that form later. Our modeling results also showed that an initial high-amplitude undulation retains its location after growth and folding. Therefore, mechanical models are able to explain the mechanisms of the formation of stable patterns of primary sulci and high variations of minor sulci across individuals^113,116,117^. In addition, there are extensive studies that emphasize the importance of sulcal pits in the normal development^118^ and function^119,120^ of the human brain.

### Effect of axonal connectivity in the folding morphology

The results of this study showed that axonal fiber bundles, after the growth and folding of the models, settle mainly in gyri rather than sulci. This observation agrees with the imaging studies that show the concentration of axonal fibers is greater in gyri than in sulci^2,43^. The density of axonal fibers in gyri is higher than in other locations, even for primates other than humans, such as macaques and chimpanzees^43,73^. We have previously shown that the axonal bundles have lower energy under tension than compression because the system tends to reach a lower energy^2^. The histogram and image of axonal fiber bundles in tension in Fig. 4a, b show a significant fraction of fibers (more than 80%) are under tension after development. This has also been observed in in *vivo* studies^121,122^. Being in gyri puts axonal bundles under tension, which is consistent with the experimental observations^123–128^. Because we considered only straight axonal fibers in our model, being under tension implies that the fibers are stretched along the radial direction. It was shown that an increase in the amplitude of positive initial undulations increases the density of axonal fibers in gyri. However, the number of undulations and concentration areas have no effect on the density of the axonal fiber bundles in gyri. Our results also are consistent with the results of a continuum model for brain development that suggests the anisotropy of the white matter induced by the axonal fibers breaks the symmetry of regular folding patterns^14,16^.

Our statistical analysis showed that LGI decreased in the presence of dense axonal fiber bundles. This observation might be due to the change in the material properties of the underlying substrate when there is a dense area of axonal fibers. The axonal fibers are approximately seven times stiffer than the ECM white matter. Therefore, locations with dense fibers form a stiffer substrate for the folding of the cortex. It is well known that an increase in the stiffness of substrate in a compressed (or heterogeneously growing) bilayer system will produce larger folds with higher wavelength^129,130^, which accordingly decreases the LGI. It should be mentioned that this observation is in the absence of the towed growth in the axonal fiber bundles. Including the towed growth concept might change the result, although the concept of the towed growth inside the ECM is not clear yet. Our results show a correlation between LGI as a representative of gyrification and density of axonal fiber bundles as a representative of brain connectivity. This observation might explain the reported correlation between abnormal gyrification and dysconnectivity in brain neurodevelopmental disorders^131–136^. A connection between abnormal folding patterns (surface morphology) of the brain and underlying connectivity has been observed in several brain disorders, such as ASD^48–52^, schizophrenia^53–58^, bipolar disorder^59,60^, as well as alteration in both domains in polymicrogyria^61–63^ and lissencephaly^64,65^. Research, including this study, suggests that axon maturation plays a critical role in gyrification^14,15,17,19,99,137^. Considering these facts, several studies support the hypothesis that brain abnormalities, such as ASD, are neurodevelopmental disconnection syndromes correlated with the different formations of fiber pathways that correlate with cortical gyrification^138–140^. In a recent cross-sectional study, the correlation between gray matter neuroanatomy and white matter connectivity in male adults with normal ASD brains was analyzed using a combined structural magnetic resonance imaging and diffusion tensor imaging study^51^. The study showed a significant difference in the local gyrification and mean diffusivity of fiber tracts in individuals with ASD compared to a normal brain. Another study using tractography and tract-based spatial statistics (TBSS) showed that the left prefrontal gyrification is negatively correlated to radial diffusivity in the forceps minor fiber tract in participants with ASD, which implies reduced cortical gyrification is connected to reduced connectivity^48^. It was also shown that there is a positive correlation between LGI and local connectivity in four fiber clusters in ASD brains by revealing that decreased gyrification is linked to increased local connectivity and reduced distant connectivity^49^. In agreement with the image-based studies, our study reveals a connection between gyrification (surface morphology) and connectivity. Therefore, this study establishes a foundation to explain the mechanics and mechanism of the gyrification-connectivity, which has not yet been thoroughly studied.

### 3-hinge gyral folds and concept of variability and regularity in folding patterns

**Figure 13:**
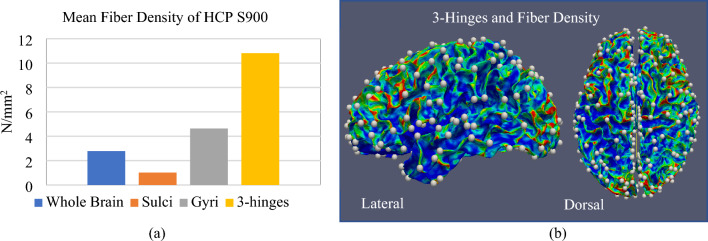
Relation between the density of axonal fibers and morphology of the brain. (**a**) Mean fiber density of HCP S900^149^ in sulci, gyri, whole brain, and 3-hinge folds. The fiber density in 3-hinge folds is even greater than typical gyri. (**b**) The density map of an example brain including the detected 3-hinge folds patterns. The locations including 3-hinges folds have greater fiber density than other locations. HCP S900 data includes 900 healthy brain data. The processing and the tractographic reconstruction from the DWI (Diffusion-Weighted Imaging) data were carried out using functions from the MRtrix3 software package^150^. For each subject, 5 × 10^4^ fiber tracts were reconstructed. The fiber density of all individuals in the HCP S900 dataset was calculated and then the mean fiber densities of the whole brain, sulci, gyri, and 3-hinges were counted.

In addition to the location of gyri and sulci, the geometrical undulations and axonal fiber bundles control the formation of 3-hinge folds in a developing brain. The locations with positive initial undulations and dense axonal fiber bundles most likely form 3-hinge folds after folding. This observation is in agreement with imaging studies that show the axonal fiber density is greater in the location of 3-hinge folds even than typical gyri in a matured brain^68^. Figure 13 shows the relation between the density of axonal fibers and the morphology of the brain. The fiber density was determined by calculating the number of fibers’ tips present in a given unit of surface area. Increasing the amplitude of undulations raises the chance of having a 3-hinge pair in all conditions. However, the number of undulations and concentration areas of axonal fibers have no considerable effect on the formation of 3-hinges.

**Figure 14:**
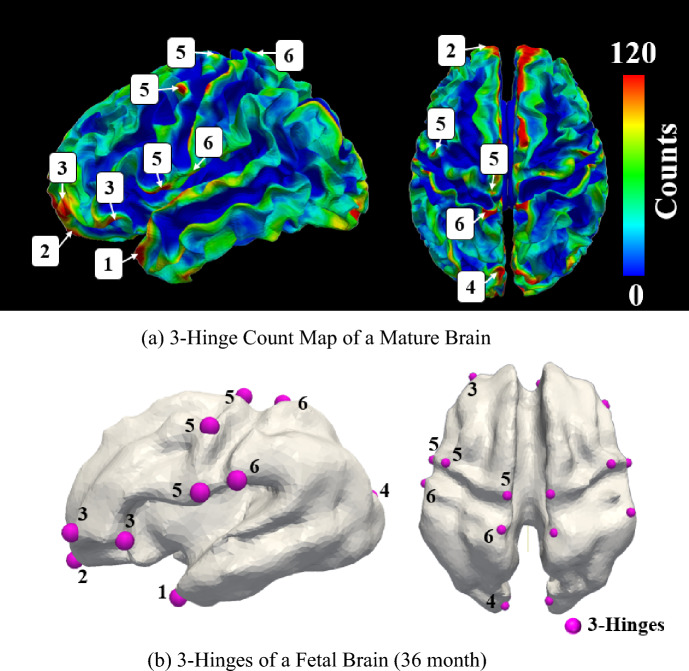
Effect of initial undulation on the formation of consistent 3-hinges. (**a**) Identified 3-hinges in a mature brain. The identified regular 3-hinges are located at 1. Temporal lobe, (**2**). Frontal lobe, 3. Rostral Middle Frontal, 4. Lateral Occipital, and 5. Precentral site. (**b**) Traced back of the associated locations with consistent 3-hinges at the fetal stage. The locations are in 1. Temporal lobe, 2. Frontal lobe, 3. Rostral Middle Frontal, 4. Lateral Occipital, 5. Precentral, and 6. Postcentral, respectively.

The results also show that in the absence of dense axonal fiber bundles or high amplitude initial undulations, DTG produces only different shapes of 3-hinges in random locations. In contrast, in the presence of dense axonal fibers or when there are high-amplitude initial undulations, the location and shape of 3-hinges are mainly consistent and preserved. This observation might explain the concept of variability and regularity of the folding patterns in the individual human brains. Despite a huge variability of folding patterns in the human brain^141–143^, there are still identified consistent 3-hinges across individual human brains^73^. The global and intense variability of folding patterns stems from the fact that all individual brains at the fetal stage have a unique initial geometry. Our models, which all feature different initial geometries, show that they can form distinct patterns after folding. As previously shown, imperfections in geometry, boundary conditions, and loading types have impacts on secondary cortical folds^42^, which is a possible factor to elucidate the complex and unique cortical morphology that forms during brain development^144^. However, consistent 3-hinge folds locations identified by imaging studies, show that their initial locations at the fetal stage have high amplitude initial undulations or areas with a high density of fibers. Figure 14a identifies several consistent 3-hinge patterns in the mature human brain. Tracing back the locations of those consistent 3-hinges shows that they have positive initial undulations at the fetal stage, Fig. 14b. The modeling findings agree very well with the imaging observations. The initial geometry, which includes positive and negative undulations and heterogeneous distribution of axonal fibers, synergistically determines the final folding patterns of the developing brain. Therefore, we hypothesize that the observed intertwined abnormal cortical morphology and axonal dysconnectivity in brain disorders such as ASD are rooted in the fetal brain development stage, even if ASD symptoms do not appear until infancy or later stages.

### Limitations and future work

In this study, similar to other computational simulations, there are multiple inherent simplifications and limitations. Direct experimental studies have shown that axonal fibers show a chronicle growth in response to a tensile force. This growth is called towed growth and has been used in brain folding models with simple geometries^14,20,121,145^. However, the complex behavior of axonal fibers inside the ECM is not well understood. We have recently quantified the hyperelastic mechanical properties of axonal fibers inside the ECM using bulk tissue material properties, micromechanical modeling, and inverse multiobjective optimization^91^. We have used those optimized material properties in the models; however, the intricate behavior of growing axonal fibers inside the white matter ECM during gyrification needs further research.

In this study, we deliberately employed a homogeneous tangential growth model in the cortical plate and assumed a uniform thickness. This decision was made to simplify the complexity of the models by reducing the number of parameters involved. However, it is important to acknowledge that in reality, the cortical plate is a nonuniform structure, exhibiting variations in thickness across different anatomical sites, and undergoes a heterogeneous growth process. Recent studies have shed light on the heterogeneity of growth patterns in the cortical plate, emphasizing the importance of considering regional variations. These findings highlight the need for a more comprehensive understanding of the dynamic changes occurring during brain development^28,85,86^. While the focus of our study was not specifically on the investigation of heterogeneous growth and its contribution, the inclusion of this factor could enhance the comprehensiveness of future models.

The consistent appearance of similar folds in all directions in our models can be attributed to the utilization of a perfectly spherical model. In such a model, the symmetrical considerations do not favor any specific folding direction, resulting in folds that exhibit similarity to one another. However, in the case of a smooth fetal brain, which possesses an irregular shape with preexisting prominent primary folds, the variation of curvature of the initial shape and the presence of significant undulations guide the folding process to yield distinct and nonuniform folds. Previous studies have elucidated the influence of the initial shape on the size and orientation of folds, emphasizing that the initial shape can significantly impact the folding patterns^36,146^. For example, it has been shown that longer brains tend to fold more longitudinally than radially^36^.

In this explanatory study, the spherical initial shape with multiple surface undulations was used to mimic the morphology of the human brain before folding. However, MRIs of fetal brains show they have an irregular shape. To be more precise, the initial geometry of the fetal brain should be reconstructed from MRI data to utilize a cortical plate of nonuniform thickness. Additionally, the distribution of axonal fiber bundles should be reconstructed from DTI fiber tractography of the fetal brains. Therefore, the next phase of our study will be the reconstruction of the initial geometry of the models from fetal MRIs and axonal fiber bundles from tractography of DTIs.

At the end, we acknowledge that the knowledge of forces^6,65,112^ alone cannot fully provide a cellular developmental interpretation of cortical folding, and understanding this complex process requires identifying the key cells involved in the formation of gyri and sulci and comprehending how these cells deform the tissue. In future studies, the demarcation between the cortical plate and the intermediate and ventricular zones^147^ should be well-defined due to the ongoing migration of neurons. This migration process exhibits spatial variability, resulting in a gradient of maturity across the entire brain. Furthermore, within the cortex, localized differential expansion occurs as a result of varying concentrations of basal radial glial fibers, which are genetically determined and contribute to the formation of gyri and sulci^86,148^. In essence, a comprehensive understanding of cerebral cortical folding necessitates examining the interplay between cells and forces, as neither factor alone is sufficient to fully comprehend this intricate process.

## Conclusion

In this study, we investigated how the interplay and hierarchy of initial geometrical irregularities, tangential differential growth in the cortical plate, and axonal connectivity affect the formation and regulation of brain folding patterns. Our results show a correlation between the location of gyri and sulci after folding and the location of initial undulations and density of axonal fiber bundles before growth. In locations with a uniform growth profile, the location of gyri and sulci is controlled by axonal fibers. The results showed that differential tangential growth is the inducer of folding, and in a hierarchical order, high-amplitude initial undulations in the cortex and axonal fibers in the substrate regulate folding patterns. After folding, the locations with dense axonal fibers settle in gyri rather than sulci. In addition, the locations of 3-hinge shapes are strongly correlated with the locations of the initial positive undulations and dense axonal fiber bundles. A major finding of this study is that there is a strong correlation between local gyrification index as a representative of the surface morphology and density of axonal fiber bundles as a representative of brain connectivity. Given that previous imaging studies attest to the correlation between connectivity and gyrification in brain disorders such as ASD, schizophrenia, and bipolar disorder, this research may shed light on the relationship between connectivity disruption and structural discrepancies that are accompanied by brain development disorders from a mechanical point of view.

### Supplementary Information

Supplementary Information.

## Data Availability

The data that support the findings of this study are available from the corresponding author upon reasonable request.

## References

[1] Vasung L (2016). Quantitative and qualitative analysis of transient fetal compartments during prenatal human brain development. Front. Neuroanat..

[2] Chavoshnejad P (2021). Role of axonal fibers in the cortical folding patterns: A tale of variability and regularity. Brain Multiphys..

[3] Alenyà M (2022). Computational pipeline for the generation and validation of patient-specific mechanical models of brain development. Brain Multiphys..

[4] Kroenke, C. D. & Bayly, P. V. How forces fold the cerebral cortex. *J. Neurosci*. **38**(4), 767–775. 10.1523/JNEUROSCI.1105-17.2017 (2018). 10.1523/JNEUROSCI.1105-17.2017 PMC5783962 29367287

[5] Dubois J (2008). Mapping the early cortical folding process in the preterm newborn brain. Cereb. Cortex N. Y. N.

[6] Fernández V, Llinares-Benadero C, Borrell V (2016). Cerebral cortex expansion and folding: What have we learned?. EMBO J..

[7] Guerrini R, Dobyns WB, Barkovich AJ (2008). Abnormal development of the human cerebral cortex: Genetics, functional consequences and treatment options. Trends Neurosci..

[8] Korotcova L (2015). Prolonged white matter inflammation after cardiopulmonary bypass and circulatory arrest in a juvenile porcine model. Ann. Thorac. Surg..

[9] Chenin L (2017). Cortical and subcortical functional neuroanatomy for low-grade glioma surgery. Neurochirurgie.

[10] Choi S-H (2021). Track-density ratio mapping with fiber types in the cerebral cortex using diffusion-weighted MRI. Front. Neuroanat..

[11] Jakab A (2014). Fetal functional imaging portrays heterogeneous development of emerging human brain networks. Front. Hum. Neurosci..

[12] Wakana S, Jiang H, Nagae-Poetscher LM, van Zijl PCM, Mori S (2004). Fiber tract–based atlas of human white matter anatomy. Radiology.

[13] Ouyang M, Dubois J, Yu Q, Mukherjee P, Huang H (2019). Delineation of early brain development from fetuses to infants with diffusion MRI and beyond. Neuroimage.

[14] Holland MA, Miller KE, Kuhl E (2015). Emerging brain morphologies from axonal elongation. Ann. Biomed. Eng..

[15] Razavi MJ (2017). Radial structure scaffolds convolution patterns of developing cerebral cortex. Front. Comput. Neurosci..

[16] Garcia KE, Wang X, Kroenke CD (2021). A model of tension-induced fiber growth predicts white matter organization during brain folding. Nat. Commun..

[17] Essen DCV (1997). A tension-based theory of morphogenesis and compact wiring in the central nervous system. Nature.

[18] Wang S, Saito K, Kawasaki H, Holland MA (2022). Orchestrated neuronal migration and cortical folding: A computational and experimental study. PLOS Comput. Biol..

[19] Xu G (2010). Axons pull on the brain, but tension does not drive cortical folding. J. Biomech. Eng..

[20] Goriely, A., Budday, S. & Kuhl, E. Neuromechanics. In *Advances in Applied Mechanics* (ed. Bordas, S. P. A.) vol. 48 79–139 (Elsevier, 2015).

[21] Hilgetag CC, Barbas H (2006). Role of mechanical factors in the morphology of the primate cerebral cortex. PLoS Comput. Biol..

[22] Tallinen T, Chung JY, Biggins JS, Mahadevan L (2014). Gyrification from constrained cortical expansion. Proc. Natl. Acad. Sci..

[23] Bayly PV, Okamoto RJ, Xu G, Shi Y, Taber LA (2013). A cortical folding model incorporating stress-dependent growth explains gyral wavelengths and stress patterns in the developing brain. Phys. Biol..

[24] Tallinen T (2016). On the growth and form of cortical convolutions. Nat. Phys..

[25] Ronan L (2014). Differential tangential expansion as a mechanism for cortical gyrification. Cereb. Cortex N. Y. N.

[26] Razavi MJ, Zhang T, Liu T, Wang X (2015). Cortical folding pattern and its consistency induced by biological growth. Sci. Rep..

[27] Zhang T (2017). Mechanisms of circumferential gyral convolution in primate brains. J. Comput. Neurosci..

[28] Rash BG, Arellano JI, Duque A, Rakic P (2023). Role of intracortical neuropil growth in the gyrification of the primate cerebral cortex. Proc. Natl. Acad. Sci..

[29] Caviness V (1975). Mechanical model of brain convolutional development. Science.

[30] Bayly PV, Taber LA, Kroenke CD (2014). Mechanical forces in cerebral cortical folding: a review of measurements and models. J. Mech. Behav. Biomed. Mater..

[31] Garcia KE, Kroenke CD, Bayly PV (2018). Mechanics of cortical folding: Stress, growth and stability. Philos. Trans. R. Soc. B Biol. Sci..

[32] Greiner A, Kaessmair S, Budday S (2021). Physical aspects of cortical folding. Soft Matter.

[33] Budday S, Raybaud C, Kuhl E (2015). A mechanical model predicts morphological abnormalities in the developing human brain. Sci. Rep..

[34] Razavi MJ, Zhang T, Li X, Liu T, Wang X (2015). Role of mechanical factors in cortical folding development. Phys. Rev. E.

[35] Toro R, Burnod Y (2005). A morphogenetic model for the development of cortical convolutions. Cereb. Cortex.

[36] Budday S, Steinmann P, Goriely A, Kuhl E (2015). Size and curvature regulate pattern selection in the mammalian brain. Extreme Mech. Lett..

[37] Campos, L. da C., Hornung, R., Gompper, G., Elgeti, J. & Caspers, S. The role of thickness inhomogeneities in hierarchical cortical folding. arXiv:200401020 *Cond-Mat Physicsphysics Q-Bio* (2020). 10.1016/j.neuroimage.2021.117779 33548459

[38] Leyva-Mendivil MF, Page A, Bressloff NW, Limbert G (2015). A mechanistic insight into the mechanical role of the stratum corneum during stretching and compression of the skin. J. Mech. Behav. Biomed. Mater..

[39] Wang L (2019). A three-layer mechanical model for the analysis of effects of pia matter on cortical folding. Eng. Comput..

[40] Wang S, Demirci N, Holland MA (2020). Numerical investigation of biomechanically coupled growth in cortical folding. Biomech. Model. Mechanobiol..

[41] Jafarabadi F, Wang S, Holland MA (2023). A numerical study on the influence of cerebrospinal fluid pressure on brain folding. J. Appl. Mech..

[42] Budday S, Steinmann P, Kuhl E (2015). Secondary instabilities modulate cortical complexity in the mammalian brain. Philos. Mag..

[43] Ge F (2018). Denser growing fiber connections induce 3-hinge gyral folding. Cereb. Cortex.

[44] Tallinen T, Biggins JS (2015). Mechanics of invagination and folding: hybridized instabilities when one soft tissue grows on another. Phys. Rev. E.

[45] Razavi MJ, Reeves M, Wang X (2017). Mechanical role of a growing solid tumor on cortical folding. Comput. Methods Biomech. Biomed. Eng..

[46] Garcia KE (2018). Dynamic patterns of cortical expansion during folding of the preterm human brain. Proc. Natl. Acad. Sci..

[47] Takahashi E, Folkerth RD, Galaburda AM, Grant PE (2012). Emerging cerebral connectivity in the human fetal brain: An MR tractography study. Cereb. Cortex.

[48] Bos DJ (2015). Reduced gyrification is related to reduced interhemispheric connectivity in autism spectrum disorders. J. Am. Acad. Child Adolesc. Psychiatry.

[49] Schaer M (2013). Decreased frontal gyrification correlates with altered connectivity in children with autism. Front. Hum. Neurosci..

[50] Pereira AM (2018). Differences in cortical structure and functional MRI connectivity in high functioning autism. Front. Neurol..

[51] Ecker C (2016). Relationship between cortical gyrification, white matter connectivity, and autism spectrum disorder. Cereb. Cortex.

[52] Ha S, Sohn I-J, Kim N, Sim HJ, Cheon K-A (2015). Characteristics of brains in autism spectrum disorder: Structure, function and connectivity across the lifespan. Exp. Neurobiol..

[53] White T, Hilgetag CC (2011). Gyrification and neural connectivity in schizophrenia. Dev. Psychopathol..

[54] White T, Gottesman I (2012). Brain connectivity and gyrification as endophenotypes for schizophrenia: Weight of the evidence. Curr. Top. Med. Chem..

[55] Sasabayashi D (2016). Increased frontal gyrification negatively correlates with executive function in patients with first-episode schizophrenia. Cereb. Cortex.

[56] Dauvermann MR (2012). Relationship between gyrification and functional connectivity of the prefrontal cortex in subjects at high genetic risk of schizophrenia. Curr. Pharm. Des..

[57] Schultz CC (2017). Increased white matter radial diffusivity is associated with prefrontal cortical folding deficits in schizophrenia. Psychiatry Res. Neuroimaging.

[58] Lubeiro A (2017). Biological and cognitive correlates of cortical curvature in schizophrenia. Psychiatry Res. Neuroimaging.

[59] He H (2017). Co-altered functional networks and brain structure in unmedicated patients with bipolar and major depressive disorders. Brain Struct. Funct..

[60] Scheepens DS (2020). The link between structural and functional brain abnormalities in depression: A systematic review of multimodal neuroimaging studies. Front. Psychiatry.

[61] Im K, Paldino MJ, Poduri A, Sporns O, Grant PE (2014). Altered white matter connectivity and network organization in polymicrogyria revealed by individual gyral topology-based analysis. Neuroimage.

[62] Squier W, Jansen A (2014). Polymicrogyria: Pathology, fetal origins and mechanisms. Acta Neuropathol. Commun..

[63] Stutterd CA, Leventer RJ (2014). Polymicrogyria: A common and heterogeneous malformation of cortical development. Am. J. Med. Genet. C Semin. Med. Genet..

[64] Vasung L (2019). Structural and diffusion MRI analyses with histological observations in patients with lissencephaly. Front. Cell Dev. Biol..

[65] Del-Valle-Anton L, Borrell V (2022). Folding brains: From development to disease modeling. Physiol. Rev..

[66] Courchesne E (2011). Neuron number and size in prefrontal cortex of children with autism. JAMA.

[67] Stoner R (2014). Patches of disorganization in the neocortex of children with autism. N. Engl. J. Med..

[68] Razavi MJ, Liu T, Wang X (2021). Mechanism exploration of 3-hinge gyral formation and pattern recognition. Cereb. Cortex Commun..

[69] Yu, X. *et al.* Joint analysis of gyral folding and fiber shape patterns. In *2013 IEEE 10th International Symposium on Biomedical Imaging* 85–88 (IEEE, 2013). 10.1109/ISBI.2013.6556418.

[70] Chen, H. *et al.* Evolutionarily-preserved consistent gyral folding patterns across primate brains. In *2014 IEEE 11th International Symposium on Biomedical Imaging (ISBI)* 1218–1221 (IEEE, 2014). 10.1109/ISBI.2014.6868095.

[71] Jiang X (2015). Sparse representation of HCP grayordinate data reveals novel functional architecture of cerebral cortex. Hum. Brain Mapp..

[72] Jiang X (2018). Temporal dynamics assessment of spatial overlap pattern of functional brain networks reveals novel functional architecture of cerebral cortex. IEEE Trans. Biomed. Eng..

[73] Li X (2017). Commonly preserved and species-specific gyral folding patterns across primate brains. Brain Struct. Funct..

[74] Dubois J, Dehaene-Lambertz G (2015). Fetal and postnatal development of the cortex: MRI and genetics. Brain Mapp. Encycl. Ref..

[75] Anderson AT (2016). Observation of direction-dependent mechanical properties in the human brain with multi-excitation MR elastography. J. Mech. Behav. Biomed. Mater..

[76] Rodriguez EK, Hoger A, McCulloch AD (1994). Stress-dependent finite growth in soft elastic tissues. J. Biomech..

[77] Gyrification from constrained cortical expansion. In *PNAS*. 10.1073/pnas.1406015111.

[78] The influence of biophysical parameters in a biomechanical model of cortical folding patterns. *Sci. Rep*. https://www.nature.com/articles/s41598-021-87124-y. 10.1038/s41598-021-87124-y PMC8032759 33833302

[79] On the growth and form of cortical convolutions. *Nat. Phys*. https://www.nature.com/articles/nphys3632.

[80] Hofman MA (1988). Size and shape of the cerebral cortex in mammals. II. The cortical volume. Brain. Behav. Evol..

[81] Finlay BL, Darlington RB (1995). Linked regularities in the development and evolution of mammalian brains. Science.

[82] Essen, D. C. V. Cerebral cortical folding patterns in primates: Why they vary and what they signify. In *Evolution of Nervous Systems* Vol. 4 (ed. Kaas, J. H.) 267–276. 10.1016/B0-12-370878-8/00344-X (Elsevier, 2007).

[83] Mota, B.,Herculano-Houzel, S. Cortical folding scales universally with surface area and thickness, not number of neurons. *Science* **349**(6243), 74–77. 10.1126/science.aaa9101. 10.1126/science.aaa9101 26138976

[84] Wang Z, Martin B, Weickenmeier J, Garikipati K (2021). An inverse modelling study on the local volume changes during early morphoelastic growth of the fetal human brain. Brain Multiphys..

[85] Zhang T (2016). Mechanism of consistent gyrus formation: An experimental and computational study. Sci. Rep..

[86] Borrell V, Götz M (2014). Role of radial glial cells in cerebral cortex folding. Curr. Opin. Neurobiol..

[87] Wang L, Yao J, Hu N (2019). A mechanical method of cerebral cortical folding development based on thermal expansion. Sci. Rep..

[88] Andescavage NN (2017). Complex trajectories of brain development in the healthy human fetus. Cereb. Cortex N. Y. N.

[89] Complex trajectories of brain development in the healthy human fetus—PubMed. https://pubmed.ncbi.nlm.nih.gov/27799276/. 10.1093/cercor/bhw306 PMC6074870 27799276

[90] Ramos AS, Paulino GH (2015). Convex topology optimization for hyperelastic trusses based on the ground-structure approach. Struct. Multidiscip. Optim..

[91] Chavoshnejad P, German GK, Razavi MJ (2021). Hyperelastic material properties of axonal fibers in brain white matter. Brain Multiphys..

[92] Kaster T, Sack I, Samani A (2011). Measurement of the hyperelastic properties of ex vivo brain tissue slices. J. Biomech..

[93] Garimella HT, Kraft RH (2016). Modeling the mechanics of axonal fiber tracts using the embedded finite element method: Axonal fiber mechanics using the embedded element method. Int. J. Numer. Methods Biomed. Eng..

[94] Guy J, Ellis EA, Kelley K, Hope GM (1989). Spectra of G ratio, myelin sheath thickness, and axon and fiber diameter in the guinea pig optic nerve. J. Comp. Neurol..

[95] Belytschko T, Fish J, Engelmann BE (1988). A finite element with embedded localization zones. Comput. Methods Appl. Mech. Eng..

[96] Fish J, Belytschko T (1988). Elements with embedded localization zones for large deformation problems. Comput. Struct..

[97] Garimella HT, Kraft RH (2017). Modeling the mechanics of axonal fiber tracts using the embedded finite element method. Int. J. Numer. Methods Biomed. Eng..

[98] Dean DC (2017). Mapping white matter microstructure in the one month human brain. Sci. Rep..

[99] Nie J (2012). Axonal fiber terminations concentrate on gyri. Cereb. Cortex N. Y. N.

[100] Lyu I, Kim SH, Girault JB, Gilmore JH, Styner MA (2018). A cortical shape-adaptive approach to local gyrification index. Med. Image Anal..

[101] Lyu I, Kim SH, Bullins J, Gilmore JH, Styner MA, Descoteaux M (2017). Novel local shape-adaptive gyrification index with application to brain development. Medical Image Computing and Computer Assisted Intervention—MICCAI 2017.

[102] Chen H (2017). Gyral net: A new representation of cortical folding organization. Med. Image Anal..

[103] Lyu, I., Kim, S. H., Bullins, J., Gilmore, J. H. & Styner, M. A. Novel Local Shape-Adaptive Gyrification Index with Application to Brain Development. In: *Medical Image Computing and Computer Assisted Intervention − MICCAI 2017. MICCAI 2017*. Lecture Notes in Computer Science, vol 10433. (eds Descoteaux, M. *et al.*) 10.1007/978-3-319-66182-7_4 (Springer, Cham, 2017).

[104] Zhang T (2018). Exploring 3-hinge gyral folding patterns among HCP Q3 868 human subjects. Hum. Brain Mapp..

[105] Libero LE, Schaer M, Li DD, Amaral DG, Nordahl CW (2019). A longitudinal study of local gyrification index in young boys with autism spectrum disorder. Cereb. Cortex.

[106] Schaer M (2008). A surface-based approach to quantify local cortical gyrification. IEEE Trans. Med. Imaging.

[107] Nishikuni K, Ribas GC (2013). Study of fetal and postnatal morphological development of the brain sulci: Laboratory investigation. J. Neurosurg. Pediatr..

[108] Ono M, Kubik S, Abernathey CD (1990). Atlas of the Cerebral Sulci.

[109] Chen H, Jiang T, Navab N, Pluim JPW, Viergever MA (2010). A dynamic skull model for simulation of cerebral cortex folding. Medical Image Computing and Computer-Assisted Intervention—MICCAI 2010.

[110] Smart IH, McSherry GM (1986). Gyrus formation in the cerebral cortex of the ferret. II. Description of the internal histological changes. J. Anat..

[111] Smart IH, McSherry GM (1986). Gyrus formation in the cerebral cortex in the ferret. I. Description of the external changes. J. Anat..

[112] Borrell V (2018). How cells fold the cerebral cortex. J. Neurosci..

[113] Im K, Grant PE (2019). Sulcal pits and patterns in developing human brains. Neuroimage.

[114] Lohmann G, von Cramon DY, Colchester ACF (2008). Deep sulcal landmarks provide an organizing framework for human cortical folding. Cereb. Cortex.

[115] Zhang S (2022). Gyral peaks: Novel gyral landmarks in developing macaque brains. Hum. Brain Mapp..

[116] Auzias G, Brun L, Deruelle C, Coulon O (2015). Deep sulcal landmarks: Algorithmic and conceptual improvements in the definition and extraction of sulcal pits. Neuroimage.

[117] Meng Y, Li G, Lin W, Gilmore JH, Shen D (2014). Spatial distribution and longitudinal development of deep cortical sulcal landmarks in infants. Neuroimage.

[118] Im K (2017). Quantitative folding pattern analysis of early primary sulci in human fetuses with brain abnormalities. Am. J. Neuroradiol..

[119] Cachia A (2003). A primal sketch of the cortex mean curvature: A morphogenesis based approach to study the variability of the folding patterns. IEEE Trans. Med. Imaging.

[120] Régis J (2005). “Sulcal root” generic model: A hypothesis to overcome the variability of the human cortex folding patterns. Neurol. Med. Chir. (Tokyo).

[121] Rajagopalan J, Tofangchi A, Saif MTA (2010). Drosophila neurons actively regulate axonal tension in vivo. Biophys. J..

[122] Dennerll TJ, Lamoureux P, Buxbaum RE, Heidemann SR (1989). The cytomechanics of axonal elongation and retraction. J. Cell Biol..

[123] Bray D (1979). Mechanical tension produced by nerve cells in tissue culture. J. Cell Sci..

[124] Heidemann SR, Buxbaum RE (1994). Mechanical tension as a regulator of axonal development. Neurotoxicology.

[125] Pfister BJ, Iwata A, Meaney DF, Smith DH (2004). Extreme stretch growth of integrated axons. J. Neurosci. Off. J. Soc. Neurosci..

[126] Suter DM, Miller KE (2011). The emerging role of forces in axonal elongation. Prog. Neurobiol..

[127] Mechanical tension contributes to clustering of neurotransmitter vesicles at presynaptic terminals. In *PNAS*. 10.1073/pnas.0901867106. 10.1073/pnas.0901867106 PMC2713391 19620718

[128] The mechanical control of nervous system development | Development | The Company of Biologists. https://journals.biologists.com/dev/article/140/15/3069/45743/The-mechanical-control-of-nervous-system. 10.1242/dev.079145 23861056

[129] Chen Y (2021). Post-wrinkling behaviors of a bilayer on a soft substrate. Int. J. Solids Struct..

[130] Holland MA, Li B, Feng XQ, Kuhl E (2017). Instabilities of soft films on compliant substrates. J. Mech. Phys. Solids.

[131] Dajani DR, Uddin LQ (2016). Local brain connectivity across development in autism spectrum disorder: A cross-sectional investigation. Autism Res..

[132] Müller R-A, Fishman I (2018). Brain connectivity and neuroimaging of social networks in autism. Trends Cogn. Sci..

[133] Alotaibi N, Maharatna K (2021). Classification of autism spectrum disorder from EEG-based functional brain connectivity analysis. Neural Comput..

[134] Ribeiro AH, Vidal MC, Sato JR, Fujita A (2021). Granger causality among graphs and application to functional brain connectivity in autism spectrum disorder. Entropy.

[135] Belmonte MK (2004). Autism and abnormal development of brain connectivity. J. Neurosci..

[136] Fornito A, Harrison B (2012). Brain connectivity and mental illness. Front. Psychiatry.

[137] Sur M, Rubenstein JLR (2005). Patterning and plasticity of the cerebral cortex. Science.

[138] Courchesne E, Pierce K (2005). Brain overgrowth in autism during a critical time in development: Implications for frontal pyramidal neuron and interneuron development and connectivity. Int. J. Dev. Neurosci..

[139] Geschwind DH, Levitt P (2007). Autism spectrum disorders: Developmental disconnection syndromes. Curr. Opin. Neurobiol..

[140] Differences in white matter fiber tract development present from 6 to 24 months in infants with autism. *Am. J. Psychiatry*. 10.1176/appi.ajp.2011.11091447. 10.1176/appi.ajp.2011.11091447 PMC3377782 22362397

[141] Van Essen DC, Drury HA, Joshi S, Miller MI (1998). Functional and structural mapping of human cerebral cortex: Solutions are in the surfaces. Proc. Natl. Acad. Sci..

[142] Fischl B, Sereno MI, Dale AM (1999). Cortical surface-based analysis. Neuroimage.

[143] Liu T, Shen D, Davatzikos C (2004). Deformable registration of cortical structures via hybrid volumetric and surface warping. Neuroimage.

[144] Naidich TP, Castillo M, Cha S, Smirniotopoulos JG (2012). Imaging of the Brain: Expert Radiology Series.

[145] Bernal R, Pullarkat PA, Melo F (2007). Mechanical properties of axons. Phys. Rev. Lett..

[146] Wang L, Yao J, Hu N (2019). A mechanical method of cerebral cortical folding development based on thermal expansion. Sci. Rep..

[147] Bernal, R., Pullarkat, P. A., & Melo, F. Mechanical properties of axons. *Phys. Rev. Lett.* **99**(1), 018301. 10.1103/PhysRevLett.99.018301 (2007). 10.1103/PhysRevLett.99.018301 17678192

[148] De Juan Romero C, Borrell V (2015). Coevolution of radial glial cells and the cerebral cortex. Glia.

[149] https://www.humanconnectome.org/study/hcp-young-adult/document/900-subjects-data-release.

[150] Tournier J-D (2019). MRtrix3: A fast, flexible and open software framework for medical image processing and visualisation. Neuroimage.

