# Supplementary material for: Mechanical hierarchy in the formation and modulation of cortical folding patterns
Source: Sci Rep. 2023 Aug 14;13:13177. doi: 10.1038/s41598-023-40086-9 (PMC10425471; doi:10.1038/s41598-023-40086-9)
Supplement: Supplementary file 1 — Supplementary Information. [file 41598_2023_40086_MOESM1_ESM.docx]

**Supplemental Materials**

**Mechanical Hierarchy in the Formation and Modulation of Cortical Folding Patterns**

Poorya Chavoshnejad^a^, Liam Vallejo^a^, Songyao Zhang^b^, Yanchen Guo^c^, Weiying Dai^c^, Tuo Zhang^b^, Mir Jalil Razavi^a*^

^a^Department of Mechanical Engineering, Binghamton University, Binghamton, NY 13902, USA.

^b^Brain Decoding Research Center and School of Automation, Northwestern Polytechnical University, Xi’an, Shaanxi, 710072, China.

^c^Department of Computer Science, Binghamton University, Binghamton, NY, USA.

^*^Corresponding author, email: [mrazavi@binghamton.edu](mailto:mrazavi@binghamton.edu)

Table S1. Correlation between percentage of area in gyri/wall/sulci with positive undulations and different parameters of models.

|  | | **Area Gyri** | **Area Wall** | **Area Sulci** |
| --- | --- | --- | --- | --- |
| **Undulations** | **Pearson Correlation** | 0.04 | -0.08 | -0.02 |
|  | **P-value** | 0.34 | 0.07 | 0.97 |
| **Concentrations** | **Pearson Correlation** | 0.08 | -0.03 | 0.01 |
|  | **P-value** | 0.86 | 0.52 | 0.77 |
| **Amplitude** | **Pearson Correlation** | **0.75**** | **-0.70**** | **-0.68**** |
|  | **P-value** | **0.01 > P** | **0.01 > P** | **0.01 > P** |

Table S2. Correlation between percentage of area in gyri/wall/sulci with negative undulations and different parameters of models.

|  | | **Area Gyri** | **Area Wall** | **Area Sulci** |
| --- | --- | --- | --- | --- |
| **Undulations** | **Pearson Correlation** | 0.03 | 0.003 | -0.03 |
|  | **P-value** | 0.42 | 0.94 | 0.49 |
| **Concentrations** | **Pearson Correlation** | **0.095*** | 0.02 | -0.08 |
|  | **P-value** | **0.04** | 0.61 | 0.06 |
| **Amplitude** | **Pearson Correlation** | **-0.80**** | **-0.55**** | **0.86**** |
|  | **P-value** | **0.01 > P** | **0.01 > P** | **0.01 > P** |

Table S3. Correlation between LGI with concentrations of axonal fiber bundles/positive undulation/ negative undulation and different parameters of models.

|  | | **Gyrification Index in Concentrations** | **Gyrification Index in Positive Undulations** | **Gyrification Index in Negative Undulations** | **Average Gyrification Index** |
| --- | --- | --- | --- | --- | --- |
| **Undulations** | **Pearson Correlation** | -0.59 | 0.14 | **-0.17*** | -0.11 |
|  | **P-value** | 0.48 | 0.87 | **0.04** | 0.20 |
| **Concentrations** | **Pearson Correlation** | **0.25**** | -0.13 | -0.13 | **-0.42**** |
|  | **P-value** | **0.01>P** | 0.12 | 0.11 | **0.01>P** |
| **Amplitude** | **Pearson Correlation** | **-0.28**** | **-0.73**** | **0.87**** | **0.49**** |
|  | **P-value** | **0.01>P** | **0.01 > P** | **0.01 > P** | **0.01 > P** |


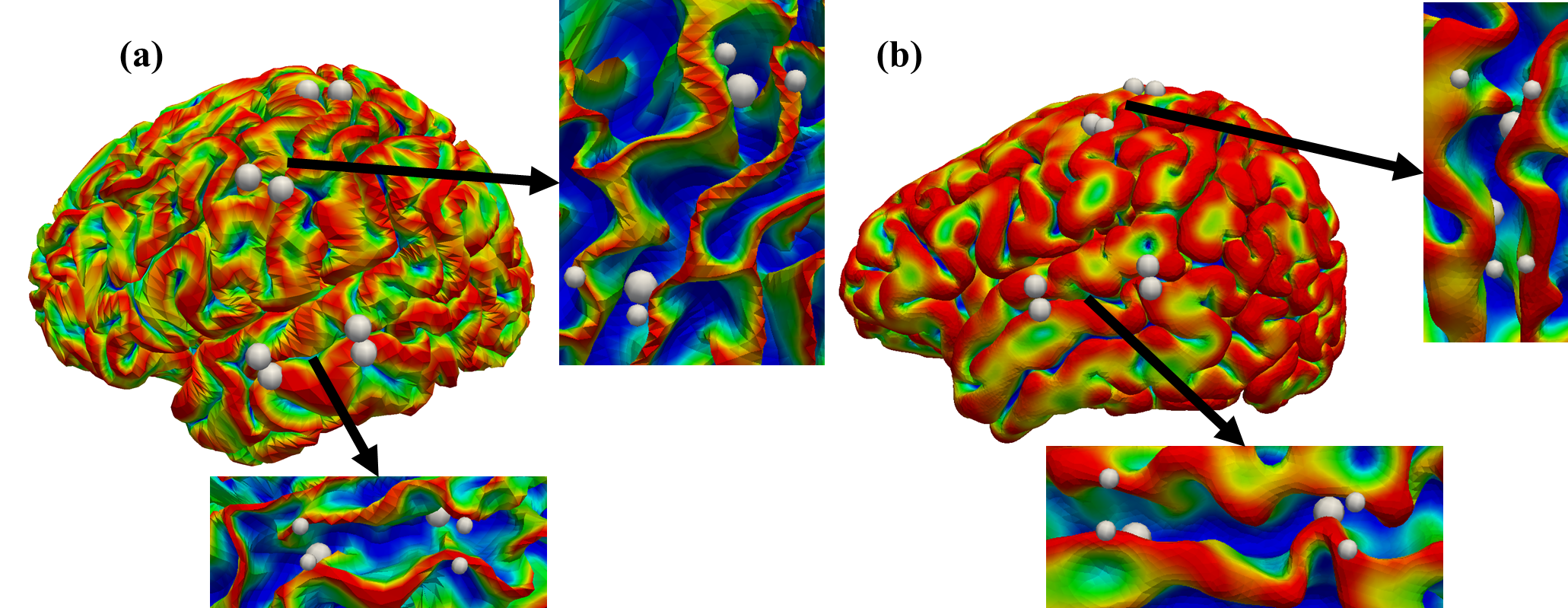


Fig. S1. An adult brain and an infant brain covered with curvature. The main images show the gray matter surface of a random subject in HCP S900 and a random subject in dHCP. The local view demonstrates the location of the selection in the sulci using the white matter surface. The white bubbles indicate the location of the sulci and gyri used for the calculations.

Table S4. The respective mean curvature of the sulci and gyri in adults and infants.

|  | **HCP** | **dHCP** |
| --- | --- | --- |
| Gyri | 0.25 | 0.40 |
| Sulci | -1.77 | -2.24 |
